# The Influence of Different Cognitive Skills on Learning Agility Among Gen Z in Established and Start-Up Companies

**DOI:** 10.3390/bs16071053

**Published:** 2026-06-24

**Authors:** Dian Palupi Restuputri, Yassierli, Ari Widyanti

**Affiliations:** 1 Department of Industrial Engineering, Bandung Institute of Technology, Bandung 40132, Indonesia; yassierli@ti.itb.ac.id (Y.); widyanti@ti.itb.ac.id (A.W.); 2 Industrial Engineering, Universitas Muhammadiyah Malang, Malang 65144, Indonesia

**Keywords:** learning agility, cognitive abilities, creativity, problem solving, generation Z, technology-driven organizations

## Abstract

Learning agility has become an essential capability for employees working in technology-driven environments characterized by rapid change and uncertainty. Despite increasing attention on learning agility, limited empirical research has examined how different levels of cognitive abilities contribute to its development, particularly among Generation Z employees. This study investigates the cognitive determinants of learning agility by distinguishing between basic cognitive abilities and high-level cognitive abilities and examining their roles across established and start-up companies. A total of 270 Generation Z employees in Indonesia participated in the study, consisting of 135 employees from established companies and 135 from start-up companies. Cognitive abilities were assessed using objective psychometric instruments, where basic cognitive abilities (reasoning, memory, attention, coordination, and perception) were measured using CogniFit, while high-level cognitive abilities were assessed through the Divergent Association Task (DAT) for creativity, the Watson–Glaser Critical Thinking Appraisal for critical thinking, and the FourSight framework for problem-solving. Learning agility was measured using a multidimensional behavioral scale. Data were analyzed using Partial Least Squares Structural Equation Modeling (PLS-SEM). The results show that higher-order cognitive abilities play a more prominent role in shaping learning agility than basic cognitive abilities. Creativity and problem solving consistently demonstrate significant positive relationships with learning agility across organizational contexts, while reasoning, critical thinking, and perception show context-dependent effects across organizational environments. These findings suggest that learning agility is primarily driven by generative and evaluative cognitive processes rather than by basic cognitive efficiency alone. The study contributes to a deeper understanding of the cognitive architecture of learning agility and provides insights for organizations seeking to develop adaptive talent in rapidly evolving technological environments.

## 1. Introduction

Agility has increasingly been recognized as a critical capability that enables organizations and individuals to respond effectively to rapid changes, uncertainty, and unexpected disruptions. Rather than functioning as a complementary skill, agility plays a central role in sustaining performance, supporting timely decision-making, and facilitating continuous adaptation. This capability becomes particularly essential as business environments grow more volatile, uncertain, complex, and ambiguous (VUCA), conditions that challenge organizational stability and long-term competitiveness (Carayannis et al., 2025). The COVID-19 pandemic further demonstrated how sudden shocks can disrupt entire industries, underscoring the importance of digital maturity and organizational flexibility (Kahveci et al., 2025). Within such contexts, agility is no longer optional but a fundamental requirement for organizational resilience and sustainable success (Kumkale, 2022).

Agility has been discussed in several forms, including organizational agility, strategic agility, workforce agility, operational agility, and digital agility. Organizational agility refers to the capacity of an organization to adjust its structures, processes, and resources in response to environmental change, while strategic agility emphasizes the ability to redirect strategic priorities when market or technological conditions shift. Workforce agility concerns employees’ flexibility in performing changing roles and tasks, whereas operational agility focuses on the responsiveness of work processes and service delivery. Digital agility is related to the ability to adopt and use digital technologies to support rapid adaptation. Among these different forms, learning agility is particularly important at the individual level because it explains how employees learn from experience and apply that learning effectively to new and unfamiliar situations (De Meuse et al., 2010). It encompasses openness to new information, willingness to experiment, and the ability to adjust strategies under uncertain conditions (Nayak & Malik, 2024). Prior studies indicate that individuals with strong learning agility are better able to navigate complexity, make informed decisions with limited information, and generate innovative solutions in ambiguous environments (Syamsir et al., 2025). At the organizational level, fostering agility requires flattening hierarchies, promoting continuous learning, and cultivating adaptability as a shared mindset across employees (Jaafar et al., 2026).

Learning agility is closely linked to cognitive abilities, as these skills provide the fundamental mechanisms for information processing, reasoning, memory, and problem-solving (DeRue et al., 2012; Hezlett & Kuncel, 2012). Cognitive abilities can be conceptually distinguished into two hierarchical levels: basic cognitive abilities and high-level cognitive abilities. Basic cognitive abilities refer to fundamental mental processes that support information processing and task execution, including attention, perception, memory, and coordination (Klahr & Wallace, 2022). These functions enable individuals to encode, store, and retrieve information efficiently, forming the foundation for more complex cognitive operations. In contrast, high-level cognitive abilities involve advanced mental processes that enable individuals to interpret, evaluate, and transform information into meaningful insights. These include critical thinking, creativity, and complex problem-solving, which support reasoning, idea generation, and decision-making in unfamiliar or dynamic situations (Dwyer et al., 2025). Together, these higher-order processes extend beyond basic information processing by enabling individuals to adapt, innovate, and respond effectively to complexity.

High-level cognitive abilities, such as critical thinking (Yusuf et al., 2024), creativity (Paz-Baruch & Maor, 2023), and complex problem-solving (Promma et al., 2025), complement basic cognitive skills including attention, perception, and coordination (Pratama et al., 2017). Together, these capabilities contribute not only to productivity but also to innovation and effectiveness when dealing with the complexity of emerging technologies. Prior research demonstrates that cognitive abilities strengthen working memory, accelerate learning processes, and enhance adaptability in demanding environments (Fuchs et al., 2023). The Trilogy of Mind framework, which integrates cognitive, affective, and conative dimensions, further explains how learning and adaptability are shaped by the interaction of thought processes, emotions, and motivation (Sudha & Premkumar, 2025). Accordingly, cognitive abilities enable individuals not only to acquire new knowledge but also to manage mental workload and make sound decisions in complex tasks (Frazier et al., 2022).

Generation Z employees represent a particularly relevant demographic in this discussion. As digital natives, they have grown up immersed in technology-rich environments and are generally familiar with digital platforms, rapid information flows, and technology-mediated work systems. This constant exposure to digital tools is often associated with technological confidence and multitasking behavior. Despite these characteristics, empirical evidence examining their cognitive capabilities and learning agility remains limited (De Meuse, 2017). Technology-intensive workplaces increasingly demand not only digital literacy but also deeper cognitive processing, sustained attention, and continuous learning. Research further indicates that young employees must develop transferable competencies, including teamwork, adaptability, and problem-solving, to function effectively in dynamic and complex work settings (Tushar & Sooraksa, 2023).

At the same time, constant interaction with fast-paced digital information may present challenges for sustained concentration and deeper analytical processing. Frequent task switching and surface-level engagement can potentially hinder the development of higher-order cognitive skills required for complex decision-making (Jose et al., 2025). Although learning agility is often associated with younger employees, limited empirical studies have systematically examined how specific cognitive abilities support agile learning and performance among Generation Z (Nayak & Malik, 2024). Understanding these relationships is therefore necessary to clarify how young employees adapt to demanding digital workplaces.

Despite increasing recognition of learning agility as a critical capability in modern organizations, prior research has largely examined cognitive abilities and learning agility as separate constructs. Limited attention has been given to differentiating the roles of basic cognitive abilities and higher-order cognitive processes in shaping agile learning behavior, particularly within rapidly changing work environments. Furthermore, empirical evidence remains scarce regarding how organizational context may condition these relationships, leaving unclear whether different workplace structures impose distinct cognitive demands that influence the development and expression of learning agility among young employees.

Work environment characteristics may further influence how cognitive abilities translate into learning agility and work performance. This study therefore considers two distinct types of technology-oriented workplaces: established companies and start-up companies. Established technology-intensive organizations typically operate with more formalized structures, specialized roles, and standardized procedures that emphasize analytical depth, sustained attention, and technical precision, characteristics commonly associated with mechanistic or structured organizational systems (Han et al., 2025). In contrast, start-ups tend to adopt more organic and flexible structures, marked by rapid change, limited resources, experimentation, and iterative problem-solving, where employees frequently assume multiple responsibilities and adapt quickly to uncertainty (Frare & Beuren, 2025). These contrasting organizational designs impose distinct cognitive and behavioral demands, potentially shaping how learning agility is developed and expressed across work settings.

The originality of this study lies in moving beyond a general association between cognitive ability and learning agility by distinguishing between different levels of cognitive functioning and examining their context-specific relevance in technology-driven work environments. Rather than treating cognition as a single broad predictor, this study differentiates basic cognitive abilities, which support information processing and task execution, from high-level cognitive abilities, which enable evaluation, idea generation, and problem solving under uncertainty. In addition, the study considers organizational context as a boundary condition by comparing established companies and start-up companies. These two contexts reflect different characteristics of digital transformation: established companies tend to emphasize formalized systems, specialized roles, data-driven decision making, and technical precision, whereas start-up companies are more strongly characterized by rapid experimentation, role fluidity, resource constraints, and uncertainty. Therefore, the study does not assume that cognitive abilities are associated with learning agility uniformly across settings. Instead, it examines whether different cognitive mechanisms become more salient under different digital work conditions.

This study aims to examine the cognitive determinants of learning agility among Generation Z employees and to compare how these relationships manifest across established companies and start-up companies. More specifically, this study addresses the following research questions: RQ1: Which basic cognitive abilities are associated with learning agility among Generation Z employees? RQ2: Which high-level cognitive abilities have the strongest relationship with learning agility? RQ3: Do the relationships between cognitive abilities and learning agility differ between established companies and start-up companies? Based on the cognitive and learning agility literature, this study proposes that basic cognitive abilities are positively associated with learning agility (H1), high-level cognitive abilities are positively associated with learning agility (H2), and the strength and significance of these relationships differ across organizational contexts (H3). By addressing these questions, this study provides empirical insight into how different cognitive abilities support young employees’ adaptive learning in contemporary digital workplaces.

## 2. Theoretical Background

### 2.1. Cognitive Abilities

Cognitive abilities play a central role in shaping how individuals perceive, process, and respond to complex and uncertain work environments. In technology-intensive contexts, employees are required to manage high information loads, multitask effectively, and make decisions under time pressure, all of which place substantial demands on cognitive resources. Prior research conceptualizes cognitive abilities as encompassing a range of mental functions that support information processing, reasoning, and problem-solving, thereby enabling individuals to perform effectively in demanding situations (Ackerman et al., 2002). These abilities are particularly critical in digitally transforming workplaces, where rapid technological change requires continuous learning and frequent adaptation of existing knowledge structures.

The literature further distinguishes between basic cognitive skills and high-level cognitive abilities, each serving different yet complementary functions in adaptive performance. Basic cognitive skills, such as reasoning, memory, attention, coordination, and perception, support accurate information processing, task execution, and efficient management of mental workload (Raemdonck et al., 2014). In contrast, high-level cognitive abilities, including creativity, critical thinking, and complex problem-solving, enable abstraction, integration of diverse information, and the generation of novel solutions when facing ill-structured problems (Amouzgar, 2022; Giannakopoulos, 2012). Empirical evidence suggests that stronger cognitive abilities enhance working memory capacity, accelerate learning processes, and improve adaptability in challenging environments (Cowan, 2014). From a theoretical perspective, the Trilogy of Mind framework reinforces this view by emphasizing that adaptive performance emerges from the interaction of cognitive, affective, and conative processes, with cognition serving as a core mechanism in learning and decision-making (Sudha & Premkumar, 2025). Empirical evidence remains limited regarding how different levels of cognitive abilities jointly or differentially contribute to learning agility, especially in digitally transforming workplaces.

### 2.2. Learning Agility

Learning agility has emerged as a critical individual capability that enables employees to remain effective in dynamic and uncertain work environments. It refers to the capacity to learn from experience and subsequently apply that learning to new, unfamiliar, or complex situations (De Meuse et al., 2010). Rather than reflecting accumulated knowledge alone, learning agility emphasizes the speed and flexibility with which individuals acquire insights, modify behaviors, and transfer prior learning across contexts. Scholars describe learning-agile individuals as open to feedback, willing to experiment, and capable of adjusting strategies when facing ambiguity (J. Lee & Song, 2022). These characteristics allow employees to navigate changing demands, solve emerging problems, and sustain performance despite limited information. Although learning agility shares conceptual proximity with adaptability, resilience, and learning orientation, it is distinguished by its explicit focus on experiential learning and behavioral transfer. Consequently, learning agility has been increasingly recognized as a key predictor of employee effectiveness, innovation, and long-term career success in technology-driven workplaces (Frolova, 2025).

### 2.3. Agility and Learning Agility in the Digital Transformation Era

The increasing prevalence of volatile, uncertain, complex, and ambiguous (VUCA) conditions has reshaped how agility is conceptualized within organizational and management research. Under such conditions, traditional planning-oriented and efficiency-driven approaches are often insufficient to address rapid environmental shifts and nonlinear disruptions (Du & Chen, 2018). Agility has therefore emerged as a critical capability that enables organizations and individuals to sense changes, interpret weak signals, and respond effectively to evolving demands (Baran & Woznyj, 2020). In technology-intensive and digitally transforming contexts, agility supports organizational resilience by enhancing responsiveness, flexibility, and adaptive decision-making (Bennett & Lemoine, 2014). As digital technologies increasingly reconfigure work processes, coordination mechanisms, and skill requirements, agility has become an important foundation for sustaining performance under uncertainty (Fletcher & Griffiths, 2020).

Within this broader discourse, learning agility has gained prominence as a distinct form of agility that emphasizes adaptive learning rather than responsiveness alone. Learning agility refers to an individual’s ability to learn from experience and apply that learning effectively to new, unfamiliar, or changing situations (J. Lee & Song, 2022). Prior studies suggest that learning agility enables individuals to cope with ambiguity through experimentation, reflection, and the continuous revision of mental models (Baran & Woznyj, 2020). Learning-agile individuals are therefore better positioned to innovate, make decisions with incomplete information, and perform effectively in complex environments shaped by digital transformation (Ruyle et al., 2021). Despite its growing importance, learning agility research has largely emphasized behavioral and contextual factors, while the cognitive capacities that enable individuals to learn and adapt effectively in digitally intensive work settings remain less clearly examined.

### 2.4. Learning Agility as an Individual Capability

Learning agility is increasingly conceptualized as a critical individual-level capability that enables employees to adapt effectively in environments characterized by uncertainty, novelty, and rapid change. At its core, learning agility reflects an individual’s capacity to learn from experience and to transfer that learning across diverse and unfamiliar contexts (De Meuse et al., 2010). Individuals with high learning agility tend to exhibit openness to feedback, curiosity, and a willingness to experiment with new approaches when faced with ambiguous situations (Carmeli & Hartmann, 2024). These attributes allow learning-agile individuals to move beyond reliance on past routines and instead engage in reflective sensemaking and continuous skill development. Empirical studies suggest that such individuals are more capable of navigating complex job demands, adapting to new technologies, and maintaining effective performance when prior experience offers limited guidance (Parker & Grote, 2022).

From an organizational standpoint, learning agility is both enabled and constrained by contextual factors such as leadership style, organizational structure, and learning culture. Research indicates that flatter organizational hierarchies, decentralized decision-making, and leadership practices that encourage experimentation and reflection can strengthen individual learning agility (Antonacopoulou et al., 2019). These conditions create opportunities for employees to encounter diverse experiences, receive timely feedback, and refine their cognitive and behavioral responses. However, despite recognition of these enabling factors, existing studies often emphasize observable behaviors and contextual antecedents of learning agility while overlooking the internal cognitive processes that support adaptive learning (De Meuse et al., 2010). As a result, the literature provides limited insight into how individuals process experience, integrate new information, and update mental models, highlighting the need to examine learning agility through a more cognitively grounded lens.

### 2.5. Cognitive on Learning Agility

Learning agility is inherently rooted in cognitive processes that enable individuals to interpret experiences, extract meaningful insights, and transfer prior knowledge to unfamiliar situations. Rather than merely reflecting behavioral adjustment, learning agility emphasizes how individuals learn from experience and reapply that learning across changing contexts (Li et al., 2024). This process depends on several interrelated cognitive mechanisms, including attention to relevant cues, memory updating, reasoning, and the restructuring of mental models, which together allow experiences to be transformed into actionable knowledge. Without these mechanisms, learning remains superficial and cannot effectively support adaptation in dynamic environments (De Meuse, 2017).

Basic cognitive skills provide the foundational capacity for these learning processes, as abilities such as attention, perception, memory, reasoning, and coordination support accurate information processing, pattern recognition, and efficient management of cognitive load (Eysenck & Brysbaert, 2018). Beyond these foundational functions, higher-order cognitive abilities including critical thinking, creativity, and complex problem solving enable individuals to reinterpret experiences, evaluate alternatives, and generate novel responses when existing routines are insufficient, thereby facilitating knowledge transfer and strategic adaptation (Alkhatib, 2019). Although the theoretical link between cognition and learning agility is well established, empirical research has seldom disentangled the distinct contributions of different cognitive levels. Learning agility is often examined primarily as a behavioral or dispositional construct, leaving its cognitive foundations underexplored. Consequently, systematic investigation of which specific cognitive abilities most strongly support learning agility remains necessary, particularly in technology-intensive work settings.

### 2.6. Technology-Driven Work Environment as a Boundary Condition: Digital Transformation Characteristics in Established and Start-Up Companies

Beyond individual capabilities, the technology-driven work environment may shape how cognitive abilities translate into learning agility and work performance. Organizational theory distinguishes between more structured and formalized systems and more flexible, organic systems (Milani et al., 2024). Within a technology-driven work environment, established companies typically operate through specialized roles, standardized procedures, and clearly defined operational processes that emphasize analytical depth, sustained concentration, and technical precision (Hashai, 2018). In contrast, start-up companies commonly adopt flatter organizational structures, rapid development cycles, limited operational resources, and continuous experimentation, requiring employees to manage multiple responsibilities while adapting quickly to uncertainty and changing demands (S. Lee, 2022). These differences indicate that technology-driven work environments impose distinct cognitive and behavioral requirements, suggesting that the development and expression of learning agility may vary between established companies and start-up companies. Examining these contrasting work environments therefore provides a more comprehensive understanding of how cognitive capabilities are enacted and transformed into adaptive learning behaviors in practice.

In this study, the comparison between established companies and start-up companies is used to capture different boundary conditions within digitally transforming work environments. Established companies often reflect digital transformation through structured technological systems, formalized procedures, data-driven decision making, and specialized work roles. These characteristics may increase the relevance of analytical evaluation, critical thinking, and systematic problem solving in supporting learning agility. In contrast, start-up companies often operate under greater uncertainty, faster iteration cycles, changing business models, and less formalized role structures. These conditions may strengthen the importance of perceptual sensitivity, creativity, and iterative problem solving because employees must respond quickly to ambiguous signals and incomplete information. Accordingly, organizational context is not treated merely as a background characteristic, but as a boundary condition that may shape which cognitive abilities become most relevant for learning agility.

### 2.7. Existing Studies and Research Gap

Existing studies have highlighted the importance of cognitive abilities and learning agility in helping employees adapt to dynamic and uncertain work environments. Prior research has positioned learning agility as an important capability associated with adaptability, leadership development, and employee performance, particularly in rapidly changing organizational settings (Baran & Woznyj, 2020; De Meuse et al., 2010; Lombardo & Eichinger, 2000). At the same time, cognitive abilities such as memory, attention, reasoning, information processing, critical thinking, and problem solving have been recognized as essential factors supporting learning and work performance (Ackerman et al., 2002; Raemdonck et al., 2014; F. L. Schmidt & Hunter, 1998). Although these studies provide valuable insights, several limitations remain. Most previous studies examined cognitive ability and learning agility separately and did not specifically identify which cognitive abilities contribute most strongly to learning agility. Existing research also tends to treat cognitive ability as a general construct without distinguishing between basic cognitive abilities and higher-level cognitive abilities. In addition, learning agility research has largely focused on leadership or managerial contexts, while empirical evidence involving Generation Z employees remains limited, despite the increasing cognitive and adaptive demands faced by younger workers in digitally intensive workplaces. Previous studies have also rarely compared how cognitive determinants of learning agility differ across organizational environments, particularly between established companies and start-up companies, which possess different levels of flexibility, uncertainty, and innovation demands. Table 1 shows the summary of previous studies and the identified research gaps related to cognitive abilities and learning agility. Therefore, this study aims to examine the cognitive determinants of learning agility among Generation Z employees and compare how these relationships manifest across established technology and start-up company contexts. By identifying which cognitive abilities most strongly support agile learning in different work settings, this study is expected to provide a more comprehensive understanding of young employees’ adaptability and performance in contemporary digital workplaces.

## 3. Methods

### 3.1. Conceptual Model

The conceptual model of this study is grounded in the premise that learning agility represents an adaptive learning capability shaped by underlying cognitive mechanisms and examined across different organizational environments (DeRue et al., 2012). Learning agility is conceptualized as the capacity of individuals to learn from experience, reinterpret knowledge, and apply learning effectively when facing new or unfamiliar situations (De Meuse et al., 2010). In contemporary work settings characterized by rapid technological change and evolving task demands, this capability becomes increasingly important as employees are required to continuously update knowledge, respond to uncertainty, and adapt to dynamic work conditions (G. Wang et al., 2024).

Within this framework, cognitive abilities are positioned as antecedents of learning agility and are differentiated into two hierarchical levels (Tan & Ho, 2023). Basic cognitive abilities represent foundational mental processes that support information processing and task execution. These include reasoning, memory, attention, coordination, and perception, which enable individuals to recognize relevant information, manage cognitive load, and maintain performance in complex work situations (Sortwell et al., 2026). These functions provide the cognitive capacity necessary for learning processes to occur, as they influence how effectively individuals encode, retain, and retrieve information from experience.

High-level cognitive abilities involve more advanced mental processes that facilitate interpretation, evaluation, and transformation of knowledge. Critical thinking, creativity, and problem-solving enable individuals to question assumptions, generate alternative perspectives, and develop new responses when existing routines are insufficient (Dondi et al., 2021). These higher-order processes are expected to play a more direct role in learning agility because they support knowledge transfer across contexts and enable adaptive responses under conditions of uncertainty (Carmeli & Hartmann, 2024). In this model, high-level cognitive abilities extend the role of basic cognition by allowing individuals not only to process information efficiently but also to reinterpret and recombine experience into new learning strategies.

The direction of the proposed model is theoretically grounded in the view that cognitive abilities provide the mental resources through which individuals process experience, evaluate information, generate alternatives, and transfer learning to unfamiliar situations. In this study, cognitive skills are therefore positioned as antecedent capabilities that support the expression of learning agility. Basic cognitive abilities provide the foundation for information processing, while high-level cognitive abilities enable interpretation, evaluation, and solution development under uncertainty. This directional specification is consistent with the dominant theoretical position in the learning agility literature. In their foundational conceptualization, DeRue et al. (2012) explicitly position cognitive abilities, particularly cognitive speed and flexibility, as individual-level antecedents that enable learning agility, defining agility itself as the capacity to rapidly assimilate information and move flexibly across ideas within and across experiences. Subsequent reviews have similarly treated cognitive capacity as an enabling condition for, rather than an outcome of, agile learning behavior (De Meuse, 2017). Moreover, empirical studies have generally found only weak to modest associations between general cognitive ability and learning agility. De Meuse et al. (2012) suggest that learning agility is not merely a reflection of cognitive capacity and that the risk of severe simultaneity between the two constructs is limited. Nevertheless, the model should be interpreted as a theoretically specified predictive model rather than as definitive evidence of causal direction.

**Figure 1:**
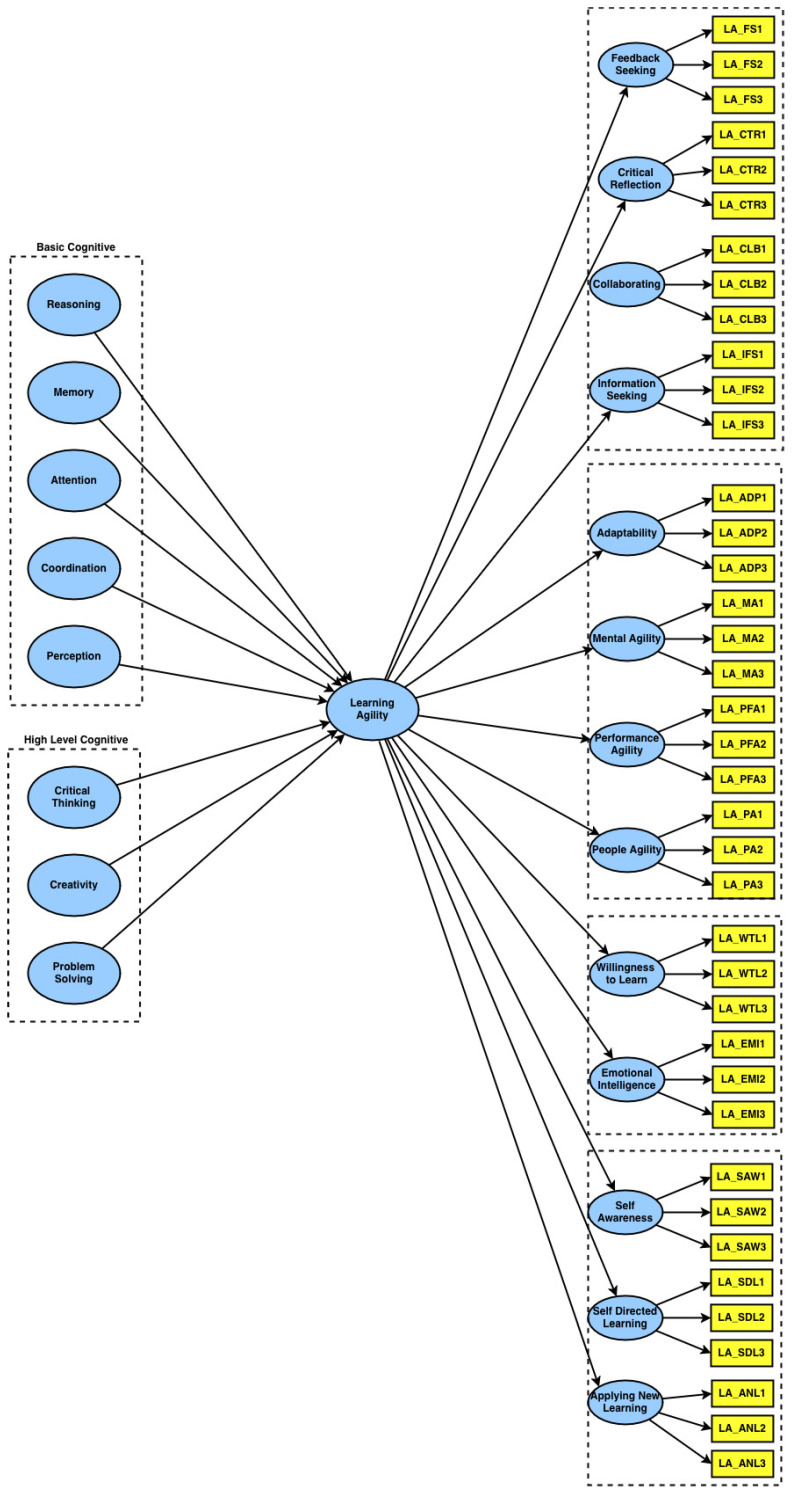
Conceptual Model. Basic cognitive abilities, including reasoning, memory, attention, coordination, and perception, are adapted from Hunt and Madhyastha (2012). High-level cognitive abilities, including critical thinking, creativity, and problem solving, are adapted from Dondi et al. (2021). The learning agility dimensions are drawn from Lombardo and Eichinger (2000), Dries and Pepermans (2012), De Meuse et al. (2012), and Hoff and Burke (2017).

The model further assumes that the relationship between cognitive abilities and learning agility may manifest differently across organizational environments. Rather than being modeled as a direct construct, organizational context is treated as a comparative analytical dimension that allows examination of how cognitive mechanisms operate under different work structures. Established and start-up companies differ in organizational structure, task flexibility, and performance expectations, which may shape how cognitive capabilities translate into learning agility behavior (Blank & Eckhardt, 2024). Examining both contexts enables a more nuanced understanding of how learning agility emerges under varying organizational conditions. The proposed conceptual model is presented in Figure 1.

As illustrated in Figure 1, the conceptual model positions basic cognitive abilities and high-level cognitive abilities as antecedent constructs influencing learning agility. Learning agility is represented as a higher-order variable reflected through thirteen dimensions, including feedback seeking, applying new learning, adaptability, mental agility, collaborating, information seeking, emotional intelligence, performance agility, people agility, willingness to learn, self-awareness, self-directed learning, and critical reflection. The figure visually depicts the hierarchical relationship in which foundational cognitive processes support higher-order cognition, and together contribute to the development of learning agility as an adaptive capability among employees.

The variables employed in this study were derived from established literature in cognitive psychology, organizational behavior, and learning agility research. Basic cognitive abilities represent foundational mental processes that support information processing and task execution, encompassing reasoning, memory, attention, coordination, and perception. High-level cognitive abilities reflect advanced cognitive processes required for interpretation, evaluation, and solution development, including creativity, critical thinking, and problem solving. Learning agility was conceptualized as a higher-order variable represented by thirteen reflective dimensions capturing individuals’ capacity to learn from experience and apply knowledge effectively in changing situations, operationalized through thirteen dimensions related to learning agility. The operational definitions, construct categorization, and supporting references for all variables used in this study are presented in Appendix A, Table A1. The selection of constructs and their conceptual definitions was guided by prior empirical studies to ensure theoretical consistency and measurement validity across cognitive and behavioral domains.

### 3.2. Subjects

The subjects of this study were Generation Z employees working in digitally intensive organizations in Indonesia. This cohort was selected due to their early-career stage and continuous exposure to digital technologies, requiring frequent learning and adaptation. Subjects were aged 20–27 years and employed full-time at the time of data collection. A total of 270 subjects were included, consisting of 135 employees from start-up companies and 135 from established companies. These two organizational contexts differ in structural characteristics, with start-ups characterized by flexibility, rapid iteration, and high uncertainty, while established companies operate with more formalized processes and specialized roles (Blank & Eckhardt, 2024). Eligibility criteria included a minimum educational level of diploma (D3) or equivalent, the ability to complete digital cognitive assessments, and active involvement in technology-enabled work processes. This dual-context sampling design enables comparative analysis of cognitive abilities and learning agility across different organizational environments.

### 3.3. Sampling Procedure and Data Collection

Participants were selected using a purposive quota sampling approach. This sampling strategy was used because the study required respondents who met specific criteria related to age, employment status, organizational context, and involvement in technology-enabled work. The inclusion criteria were: (1) being a Generation Z employee aged 20–27 years, (2) working full-time in a technology-driven organization, (3) having at least a diploma-level educational background or equivalent, (4) being actively involved in technology-enabled work processes, and (5) being able to complete digital cognitive assessments. Respondents who did not meet these criteria or who provided incomplete responses were excluded from the final dataset.

To enable comparison across organizational contexts, the sample was divided into two equal groups: 135 employees from established companies and 135 employees from start-up companies. The equal group size was determined to support balanced comparative analysis between the two organizational settings. Established companies were defined as more formalized technology-oriented organizations with structured roles and procedures, while start-up companies were defined as more flexible and innovation-oriented organizations characterized by rapid experimentation, role fluidity, and higher uncertainty.

The data collection process was conducted in several stages. First, potential respondents were screened based on the inclusion criteria. Second, eligible respondents were informed about the purpose of the study, the voluntary nature of participation, confidentiality of responses, and the general procedures involved in the assessment. Only respondents who provided informed consent were included in the study. Third, participants completed the cognitive assessments, including the CogniFit tasks for basic cognitive abilities and standardized instruments for creativity, critical thinking, and problem solving. Fourth, respondents completed the learning agility questionnaire. During the assessment process, the researchers provided instructions and clarification when needed to ensure that participants understood the task requirements. Data were checked for completeness before analysis, and incomplete responses were excluded.

The sample was not intended to represent the entire population of Generation Z employees in Indonesia statistically. Rather, it was designed to be analytically relevant to the study objective by focusing on Generation Z employees working in technology-driven organizational contexts. Therefore, the findings should be interpreted as providing evidence for the examined sample and comparable digital work environments, rather than as a generalization to all Generation Z employees across industries or regions.

To minimize potential common method bias during data collection, several procedural controls were applied. The predictor and outcome variables were not measured using the same method. Cognitive skills were assessed using cognitive ability tasks/tests, whereas learning agility was measured using a structured questionnaire. This separation of measurement methods reduced the likelihood that the observed relationships were driven mainly by common response tendencies. Respondents were also assured that their responses would remain confidential and would be used only for research purposes. They were informed that there were no right or wrong answers in the questionnaire section, which helped reduce evaluation apprehension and social desirability bias. In addition, the cognitive assessment and learning-agility questionnaire were presented as separate sections to reduce the possibility that respondents would infer the expected relationships among the study variables.

Several procedural remedies were applied during data collection to minimize the potential for common method and response biases (Podsakoff et al., 2003). First, the use of objective cognitive assessments alongside a self-reported learning agility scale created a temporal and methodological separation between the measurement of predictor and outcome variables. Second, respondents were assured of the anonymity and confidentiality of their responses and were informed that there were no right or wrong answers, in order to reduce social desirability and evaluation apprehension. Third, items were presented using clear and concise wording to reduce ambiguity and response fatigue. These procedural safeguards were applied to reduce the likelihood that the observed relationships were driven mainly by common response tendencies.

### 3.4. Instruments

#### 3.4.1. Measurement of Basic Cognitive Abilities

The measurement focused on five basic cognitive domains: reasoning, memory, attention, coordination, and perception (Lyman & Kuderer, 2023; Sung et al., 2023). Basic cognition was measured using CogniFit, a validated digital psychometric platform accessible via smartphones and computers (Embon-Magal et al., 2022). The platform includes structured tasks targeting each domain: logical inference for reasoning, short-term and working memory tasks, sustained and selective attention tests, sensorimotor coordination activities, and perception-based information processing tasks. CogniFit was selected due to its demonstrated reliability across diverse populations and its ability to generate standardized quantitative scores suitable for structural modeling.

The measurement was administered directly to subjects using smartphones. Prior to the assessment, subjects were introduced to the CogniFit application and completed a short trial session of approximately three minutes to familiarize themselves with the task interface and instructions. Once subjects indicated readiness, the cognitive assessment tasks were conducted. During the assessment process, subjects were accompanied by the researchers to ensure that instructions were clearly understood and that each task was completed according to the required procedures. Scores from each domain were aggregated and standardized to form latent variables representing basic cognitive abilities. These constructs were modeled as exogenous variables in the PLS-SEM framework to examine their influence on learning agility.

#### 3.4.2. Measurement of High-Level Cognitive Abilities

The measurement of high-level cognitive abilities focused on advanced mental processes essential for innovation, complex problem solving, and learning in technology-intensive settings. High-level cognition encompasses creativity, critical thinking, and problem-solving capabilities that enable individuals to respond effectively to novel challenges and dynamic work demands (Dumitru & Halpern, 2023; Nilimaa, 2023). These abilities complement basic cognitive functions and provide the strategic foundation for learning agility (De Meuse, 2017). Creativity was measured using the Divergent Association Task (DAT), a rapid verbal assessment of divergent thinking that requires subjects to generate semantically distant words (Olson et al., 2021). Critical thinking was assessed using the Watson–Glaser Critical Thinking Appraisal administered via the Assessment Day platform (Al-Ghadouni, 2021). This instrument evaluates argument analysis, assumption identification, deductive reasoning, decision-making, and information interpretation, capturing evaluative reasoning essential for informed decision-making in complex contexts (Thornhill-Miller et al., 2023). Problem-solving ability was measured using FourSight Online, a validated psychometric tool that assesses preferences across four cognitive styles: Clarifier, Ideator, Developer, and Implementer. This framework captures how individuals structure problems, generate ideas, refine solutions, and implement outcomes, reflecting comprehensive engagement in practical problem-solving processes (Garwood, 2020). Prior studies report strong internal reliability for this instrument in assessing higher-order cognition (Puccio & Acar, 2015). For PLS-SEM modeling, creativity, critical thinking, and problem solving were treated as latent variables representing high-level cognitive abilities. Scores were standardized to facilitate integration with learning agility.

#### 3.4.3. Measurement of Learning Agility

Learning agility was operationalized through thirteen dimensions: Adaptability, Applying New Learning, Collaborating, Emotional Intelligence, Feedback Seeking, Mental Agility, Information Seeking, People Agility, Performance Agility, Critical Reflection, Self-awareness, Self-directed Learning, and Willingness to Learn. Each dimension was measured using three indicators, yielding a total of thirty-nine indicators. To improve transparency of the measurement model, the complete list of learning agility dimensions, item codes, and indicators is provided in Appendix A, Table A2. This configuration balances measurement reliability with respondent burden while ensuring adequate construct representation for latent variable modelling (J. F. Hair et al., 2019).

Learning agility was modeled as a higher-order reflective construct because the thirteen dimensions represent interrelated manifestations of a broader adaptive learning capability. Conceptually, these dimensions reflect different behavioral expressions of the same underlying capacity to learn from experience and apply learning effectively in new or changing situations. Treating learning agility as a higher-order construct allows the analysis to preserve the multidimensional structure of the construct while estimating its overall relationship with cognitive abilities. This approach is consistent with the view that learning agility is not captured by a single behavior, but by a constellation of learning-related tendencies involving reflection, adaptability, feedback seeking, knowledge application, and willingness to learn.

The initial item pool was developed through a comprehensive literature synthesis and refined through expert validation. The experts involved in this stage consisted of Human Resource Development (HRD) practitioners and academics in the field of Industrial Engineering who evaluated the overall construct definition, conceptual clarity, and the relevance of each item in representing learning agility behaviors within contemporary work environments. The review process included the assessment of conceptual descriptions, the operational explanation of each dimension, and the suitability of item wording. Qualitative feedback from the experts was subsequently used to refine the instrument. Conceptually overlapping constructs were consolidated to enhance clarity and parsimony (J. Lee & Song, 2022). Content validity was assessed using the Item-level Content Validity Index (I-CVI), with items meeting the recommended threshold of 0.80 retained (Azli Syam et al., 2026). Items that did not meet this threshold were either revised or replaced with alternative items that better represented the intended construct. This procedure ensured that each indicator demonstrated adequate conceptual relevance prior to empirical testing. The final instrument therefore consisted of thirteen learning agility dimensions.

In the empirical stage, the measurement properties of the learning agility scale were evaluated using PLS-SEM. Convergent validity was assessed using indicator loadings, while construct reliability was evaluated through Cronbach’s alpha, composite reliability, and average variance extracted (AVE). Indicators with loadings exceeding the recommended threshold were retained, indicating adequate measurement reliability and validity. Within the PLS-SEM framework, the thirteen learning agility dimensions were specified as reflective first-order constructs representing the multidimensional structure of learning agility used in the structural analysis (J. F. Hair et al., 2019).

#### 3.4.4. Data Analysis

Before conducting structural modeling, preliminary statistical analyses were performed to compare cognitive abilities and learning agility scores between employees in start-up and established companies. Descriptive statistics were first calculated to summarize the distribution of basic cognitive abilities, high-level cognitive abilities, and learning agility scores across both groups. Data normality was assessed using the Kolmogorov–Smirnov test. The results indicated that most variables deviated from a normal distribution (*p* < 0.05); therefore, non-parametric procedures were applied. Differences between the two groups were examined using the Mann–Whitney U test. These analyses provided an initial empirical overview of whether employees in different organizational environments exhibited statistically significant differences in cognitive capabilities and learning agility profiles.

To examine the structural relationships among variables, PLS-SEM was employed. This variance-based approach is appropriate for complex research models involving multiple latent constructs, non-normal data distributions, and predictive analysis objectives (J. Hair & Alamer, 2022). Separate structural models were estimated for the start-up and established company groups to enable contextual comparison between organizational environments. This comparative approach allowed the study to examine whether the cognitive determinants of learning agility differed across two digital work contexts with distinct transformation characteristics, rather than assuming a uniform cognition–learning agility relationship across all organizations.

In the structural model, basic cognitive abilities, consisting of reasoning, memory, attention, coordination, and perception, and high-level cognitive abilities, consisting of critical thinking, creativity, and problem solving, were specified as exogenous variables. Learning agility was modeled as an endogenous reflective variable represented by thirteen behavioral dimensions. The measurement model was evaluated through indicator loadings, construct reliability, including Cronbach’s alpha and composite reliability, and average variance extracted (AVE). Discriminant validity was further assessed using the Fornell–Larcker criterion and the Heterotrait–Monotrait ratio of correlations (HTMT). The Fornell–Larcker criterion was used to examine whether the square root of AVE for each construct exceeded its correlations with other constructs, while HTMT was used as an additional assessment of discriminant validity. Multicollinearity was assessed using Variance Inflation Factor (VIF) values. Structural relationships were evaluated using bootstrapped path coefficients, coefficients of determination (R^2^), and effect sizes (f^2^).

The structural paths were interpreted according to the theoretically specified direction from cognitive abilities to learning agility. Given the cross-sectional design of the study, the analysis identifies predictive associations within the proposed model rather than establishing definitive causal effects. Potential reverse causality, in which learning agility may also contribute to the development or use of cognitive skills over time, could not be fully ruled out in the present design.

Organizational context was used as a comparative analytical dimension rather than as a formal moderating variable. This decision was made because the study focused on identifying context-specific cognitive determinants of learning agility within each organizational setting rather than testing a direct interaction effect between cognitive abilities and organizational context. Separate models for established companies and start-up companies were therefore estimated to compare the structural patterns across organizational environments. This analytical procedure enabled the identification of cognitive factors most strongly associated with learning agility within each organizational context and provided comparative evidence on how different work environments shape these relationships.

## 4. Results

### 4.1. Subjects

The sample consisted of subjects employed in start-up and established companies in Indonesia, representing technology-driven work environments. Based on company type, subjects in the start-up group were primarily drawn from digital creative industry (36%) and software development (32%), followed by financial technology (17%) and food and beverage sectors (15%), reflecting the dominance of digital service and platform-based businesses in start-up ecosystems. In contrast, subjects in the established companies were mainly employed in the consumer electronics industry (44%), telecommunications (29%), and electrical power industry (27%), indicating a stronger representation of technology-integrated manufacturing and infrastructure-based sectors. In terms of gender distribution, the start-up group consisted of 45% male and 55% female subjects, while the established companies showed a higher proportion of male subjects (58%) compared to female subjects (42%). Regarding job categories, the largest proportions of subjects in both groups were employed as software engineers, engineering personnel, and data and analytics specialists, with engineering roles more prominent in established companies and software engineering and customer-related roles more common in start-ups. With respect to educational background, the majority of subjects in both groups held a bachelor’s degree, accounting for 99% of the start-up group and 96% of the established companies group, while only a small proportion had diploma-level education.

### 4.2. Measurement of Basic Cognitive Abilities

**Figure 2:**
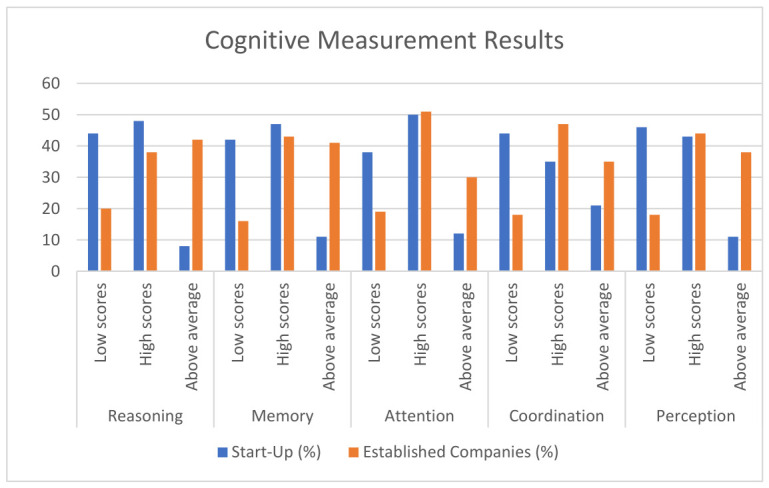
Descriptive Statistics of Basic Cognitive Abilities.

The summary of cognitive measurement results for each group is presented in Figure 2. Descriptive statistics were first analyzed to examine the distribution of basic cognitive scores across the two work environments.

### 4.3. Measurement of High-Level Cognitive Abilities

**Figure 3:**
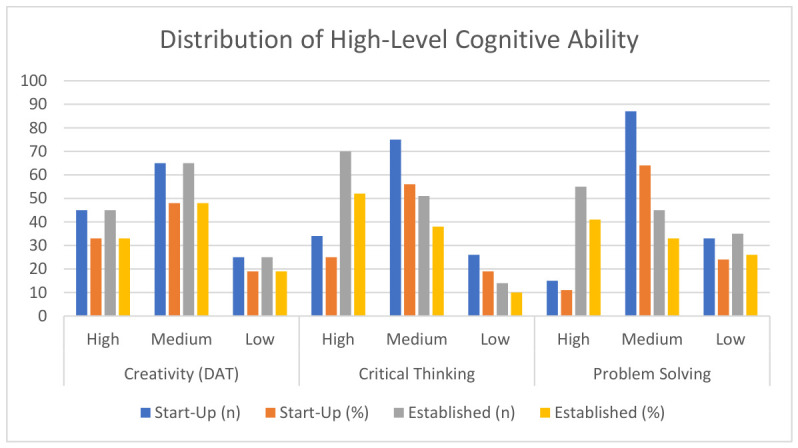
Distribution of High-Level Cognitive Ability Levels Across Work Environments.

The measurement of high-level cognitive abilities was conducted to describe the distribution of advanced cognitive processes that support learning agility in different work environments. The overall distribution of creativity, critical thinking, and problem-solving across both work environments is summarized in Figure 3.

### 4.4. Measurement of Learning Agility

The final instrument consisted of thirteen learning agility dimensions, namely Feedback Seeking, Applying New Learning, Adaptability, Mental Agility, Collaborating, Information Seeking, Emotional Intelligence, Performance Agility, People Agility, Willingness to Learn, Self-Awareness, Self-Directed Learning, and Critical Reflection. Each dimension was represented by three indicators, resulting in a total of thirty-nine measurement items designed to capture behavioral tendencies and learning-related responses among Generation Z employees. The complete indicator structure is presented in Appendix A, Table A2, to assess the content coverage of the learning agility measurement model.

#### 4.4.1. Measurement Model Evaluation

The measurement properties of the variables were evaluated using the PLS-SEM approach. The evaluation focused on assessing convergent validity, discriminant validity, and construct reliability to ensure that the measurement model adequately represents the theoretical structure of the constructs. Convergent validity was assessed using indicator loadings. The results indicate that all indicator loadings exceeded the recommended threshold of 0.70, demonstrating satisfactory indicator reliability. Discriminant validity was examined through cross-loading analysis, where each indicator showed a higher loading on its associated dimension than on other dimensions, confirming that the indicators appropriately represent their respective dimensions. Construct reliability was evaluated using Cronbach’s alpha, composite reliability, and AVE. The results show that all constructs satisfied the recommended thresholds (Cronbach’s alpha > 0.70, composite reliability > 0.70, and AVE > 0.50), indicating satisfactory internal consistency and convergent validity. These results confirm that the measurement model meets the required reliability and validity criteria and is therefore suitable for further structural model analysis.

Additional diagnostic analyses were conducted to strengthen the empirical assessment of the measurement and structural models. Discriminant validity was examined using the Fornell–Larcker criterion and the HTMT ratio. The Fornell–Larcker results showed that the square root of AVE for each construct was higher than its correlations with other constructs in the combined sample, established companies sample, and start-up companies sample. These results indicate that the constructs demonstrated adequate discriminant validity across the three datasets.

The HTMT results also supported discriminant validity. In the combined sample, all HTMT values were below 0.85, with the highest value approximately 0.830. In the start-up companies sample, all HTMT values were also below 0.85, with the highest value being approximately 0.849. In the established companies sample, most HTMT values were below 0.85, while a small number of values among closely related learning agility dimensions slightly exceeded 0.85 but remained below 0.90, with the highest value being approximately 0.876. Because these dimensions represent theoretically related manifestations of the broader learning agility construct, the results were considered acceptable and supported discriminant validity.

Multicollinearity was assessed using VIF values. In the combined sample, the VIF values for the structural paths ranged from 1.301 to 2.357. In the established companies sample, the VIF values ranged from 1.279 to 2.718, while in the start-up companies sample, the VIF values ranged from 1.258 to 2.474. All values were below the recommended threshold of 3.3, indicating that multicollinearity was not a concern in the structural model estimation. The full collinearity VIF values ranged from 1.000 to 1.950 in the combined sample, from 1.000 to 2.344 in the established companies sample, and from 1.000 to 1.944 in the start-up companies sample. Since all values were below the recommended threshold of 3.3, the results suggest that method bias was unlikely to substantially affect the model estimation. In addition, the risk of method bias was reduced by the measurement design because cognitive abilities were assessed using objective psychometric instruments, while learning agility was measured using self-reported behavioral indicators. The complete Fornell–Larcker and HTMT results are provided in the Supplementary Materials.

#### 4.4.2. Second-Order Measurement Model

After confirming the adequacy of the first-order measurement model, the second-order measurement model was evaluated to examine whether the thirteen dimensions collectively represent the higher-order variable of learning agility. The results show that the dimension loadings ranged from 0.698 to 0.817. Most dimensions met the recommended threshold of 0.70, while one loading was marginally below the threshold and was retained because it remained close to the recommended value and was theoretically important for representing the multidimensional structure of learning agility. These findings confirm that learning agility can be represented as a multidimensional higher-order construct composed of thirteen reflective dimensions. Therefore, all dimensions were retained in the model and used in the subsequent structural model analysis.

### 4.5. Cognitive Determinants of Learning Agility

Following the confirmation of the first- and second-order measurement models, the structural model was estimated to examine the influence of basic cognitive abilities and high-level cognitive abilities on learning agility. To assess robustness across organizational contexts, the analysis was conducted using three datasets: the combined sample dataset, the established companies dataset, and the start-up dataset.

Additional outer model evaluations were performed for each dataset to verify construct validity and reliability. For the combined and established companies datasets, all indicators satisfied the recommended measurement criteria, including factor loadings above 0.70, AVE values above 0.50, and Cronbach’s Alpha and Composite Reliability values above 0.70. Discriminant validity was confirmed using cross-loadings and the Fornell–Larcker criterion.

For the start-up dataset, three learning agility indicators, namely willingness to learn, self-awareness, and critical reflection, showed loading values below the threshold of 0.70 in the measurement model and were therefore removed before the final estimation. After re-estimation, the revised measurement model satisfied the required validity and reliability criteria. The removal of these three indicators in the start-up dataset was carefully considered in relation to cross-sample comparability. The indicators were removed only after they failed to meet the recommended loading threshold in the start-up measurement model, indicating that these indicators did not adequately represent the higher-order learning agility construct in this specific subgroup. Therefore, the revised start-up model was retained to ensure measurement reliability and validity within that sample. At the same time, the comparison across samples should be interpreted as a comparison of structural patterns rather than as strict metric equivalence across fully identical measurement models. The analysis focuses on whether the direction and significance of cognitive determinants differ across organizational contexts, while acknowledging that the start-up model reflects a refined measurement structure. The hypothesis testing results are presented in Table 2.

The explanatory power of the structural model was assessed using the coefficient of determination (R^2^) and adjusted coefficient of determination (Adjusted R^2^), as presented in Table 3. The results indicate that the proposed cognitive variables explained a substantial proportion of the variance in learning agility across all datasets. The highest explanatory power was observed in the established companies sample. The relatively small differences between the R^2^ and Adjusted R^2^ values across all models suggest that the explanatory capability of the models remained stable after accounting for the number of predictors included in the analysis. These findings indicate that cognitive abilities represent important determinants of learning agility, particularly within established companies, where cognitive factors appear to play a more prominent role in shaping adaptive learning behavior.

**Figure 4:**
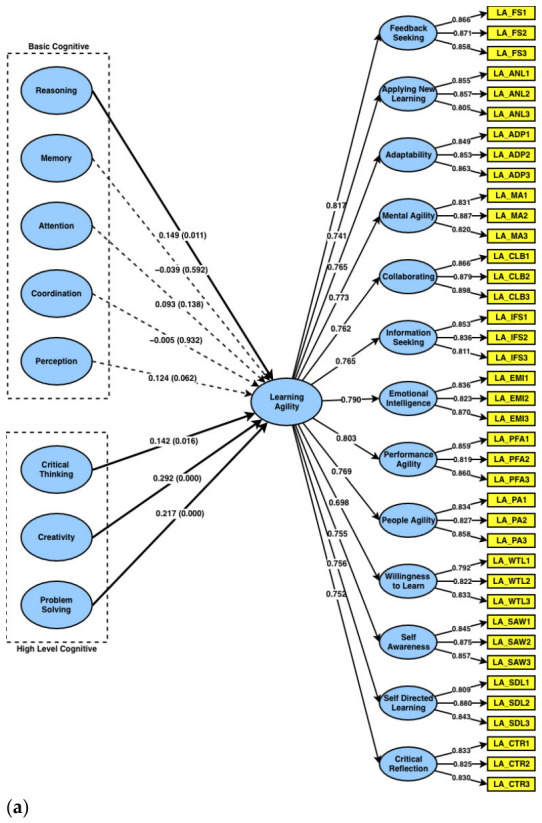

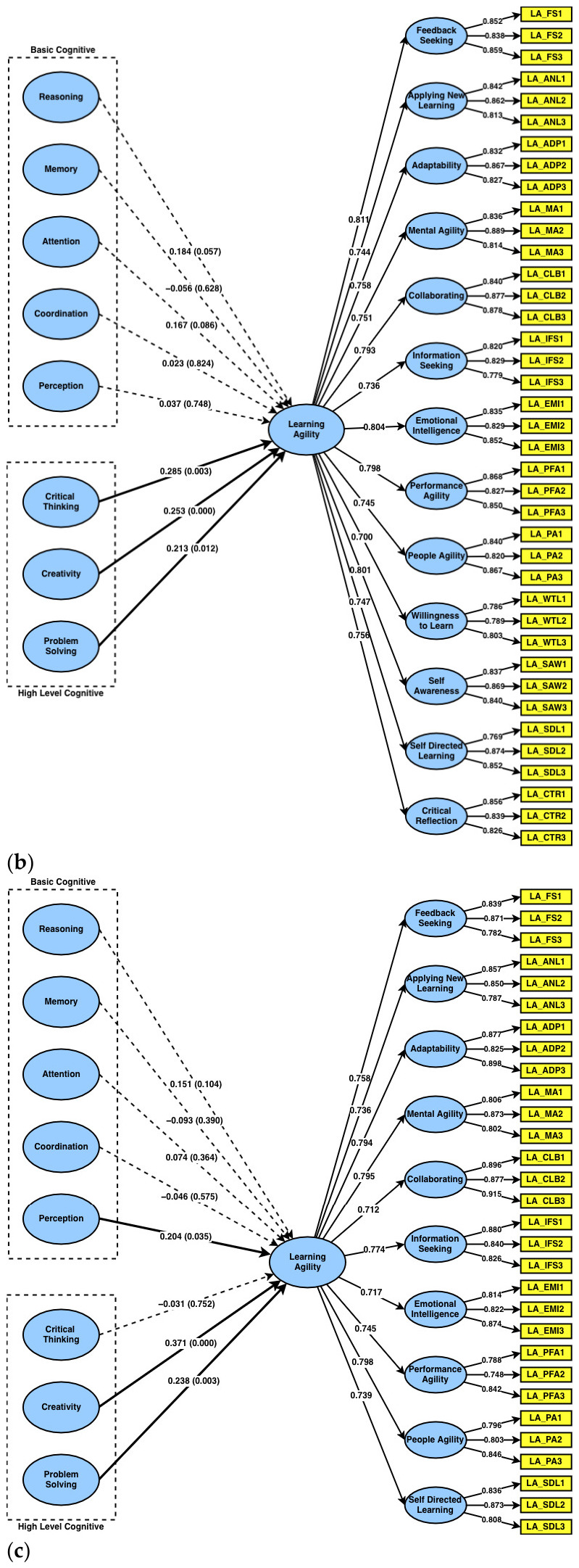
Empirical Models of Cognitive Determinants of Learning Agility: (**a**) Combined-Sample Model, (**b**) Established Companies Model, and (**c**) Start-Up Companies Model. Annotation: Solid bold lines indicate statistically significant structural relationships (*p* < 0.05), while dashed lines indicate non-significant relationships. Panel (**a**) represents the combined-sample model, Panel (**b**) represents the established companies model, and Panel (**c**) represents the start-up companies model.

Figure 4 presents the empirical models of cognitive determinants of learning agility using a consistent sample order. Panel (a) shows the combined-sample model, Panel (b) presents the established companies model, and Panel (c) shows the start-up companies model. The combined-sample model provides the overall structural pattern, while the two subgroup models illustrate how the relationships between cognitive abilities and learning agility differ across organizational contexts. In the start-up model, the displayed measurement structure reflects the revised model after the removal of indicators that did not meet the recommended loading threshold.

## 5. Discussion

### 5.1. Cognitive Determinants of Learning Agility in the Combined Sample

The findings reported in this section refer to the combined-sample model, which includes respondents from both established companies and start-up companies. This model provides an overall view of how basic cognitive abilities and high-level cognitive abilities are associated with learning agility among Generation Z employees before the results are compared across organizational contexts. In the combined sample, reasoning, critical thinking, creativity, and problem solving show significant relationships with learning agility, while memory, attention, coordination, and perception do not demonstrate significant direct effects.

The findings of this study indicate that learning agility among Generation Z employees is shaped primarily by higher-level cognitive abilities rather than by basic cognitive efficiency. In the overall model, reasoning, critical thinking, creativity, and problem solving are associated with learning agility, although their significance varies across organizational contexts. This pattern indicates that adaptive learning behavior is closely connected to cognitive processes that allow individuals to interpret, evaluate, and transform information when facing unfamiliar situations. These results contribute to the growing body of literature that views learning agility as a cognitive capability rather than solely a behavioral tendency or personality-based attribute (De Meuse, 2017; De Meuse et al., 2012). While earlier studies have often emphasized behavioral indicators such as openness to experience and willingness to learn, the present findings suggest that the cognitive processes underlying learning behavior deserve greater attention in understanding how individuals adapt to new challenges in the workplace.

Among the cognitive variables examined in this study, creativity emerges as the strongest predictor of learning agility. Creativity reflects the capacity to generate novel ideas, explore alternative perspectives, and recombine existing knowledge when confronting complex problems (Puccio et al., 2023). In organizational environments characterized by rapid technological change and evolving work demands, individuals who are able to think creatively may be better equipped to reinterpret existing knowledge and develop new approaches to emerging challenges. Creative cognition encourages experimentation and exploration, which are central mechanisms through which individuals refine their learning strategies. Employees with stronger creative capabilities may therefore engage more actively in reflective learning processes and be more willing to test new approaches when faced with unfamiliar situations.

Problem solving also demonstrates a significant positive relationship with learning agility in the overall model. Problem-solving ability represents the capacity to identify challenges, analyze possible solutions, and implement effective strategies to address complex situations (Loureiro & Pereira, 2024). In modern work environments where employees frequently encounter novel problems and ambiguous tasks, individuals with strong problem-solving skills may transform workplace challenges into opportunities for learning and development. Through systematic evaluation of possible solutions, employees are able to refine their approaches and improve their performance over time. This iterative learning process supports the development of adaptive learning behavior and strengthens learning agility.

The significant influence of reasoning further highlights the importance of analytical cognitive processes in supporting adaptive learning behavior. Reasoning enables individuals to interpret complex information, recognize patterns, and construct logical explanations when confronted with new problems (Bronkhorst et al., 2020). Individuals with stronger reasoning abilities may therefore be better equipped to transfer knowledge across contexts and apply lessons from previous experiences to new situations. This ability to restructure mental models and reinterpret information contributes directly to the development of learning agility.

In contrast, several basic cognitive abilities, including memory, attention, and coordination, do not demonstrate significant direct effects on learning agility in the overall model. These abilities are primarily associated with efficient information processing and task execution. While they play an important role in supporting general cognitive functioning, they may not directly influence the adaptive learning processes required to navigate complex and rapidly changing work environments. This finding suggests that learning agility depends less on the efficiency of basic cognitive functions and more on the ability to interpret and transform information through higher-order cognitive processes.

These findings are broadly consistent with prior learning agility research, which emphasizes the ability to learn from experience and apply that learning to novel and unfamiliar situations (De Meuse et al., 2010; De Meuse, 2017). Earlier studies have often described learning agility through behavioral characteristics such as openness to feedback, willingness to experiment, flexibility, and readiness to learn from challenging experiences. The present study extends this literature by showing that these behavioral tendencies are supported more strongly by higher-level cognitive abilities than by basic cognitive efficiency alone. In particular, the significant roles of creativity and problem solving suggest that learning-agile employees are not only able to absorb new information, but also to reinterpret experience, generate alternative responses, and develop workable solutions when existing routines are no longer sufficient. This finding also complements previous work linking cognitive ability to learning and performance by demonstrating that not all cognitive abilities contribute equally to learning agility. While memory, attention, and coordination remain important for general task execution, the ability to evaluate, restructure, and transform information appears to be more central to adaptive learning behavior in technology-driven work environments.

### 5.2. Cognitive Determinants of Learning Agility in Established Companies

The findings for established companies reveal a different pattern in the cognitive determinants of learning agility. In this context, critical thinking, creativity, and problem solving demonstrate significant positive relationships with learning agility. This configuration reflects the cognitively demanding nature of technologically sophisticated workplaces, where employees must frequently evaluate complex information and develop solutions to technical challenges.

Critical thinking plays a particularly important role in these environments because employees must analyze data, evaluate assumptions, and assess alternative explanations when addressing complex problems (Dumitru & Halpern, 2023). Established companies often operate within structured systems where decisions must be supported by logical reasoning and empirical evidence. Employees with strong critical thinking abilities may therefore be more capable of interpreting technical information accurately and adapting their learning strategies when confronting complex tasks.

Creativity remains an important determinant of learning agility even in structured technological environments. Technological innovation frequently requires employees to recombine existing knowledge in new ways and generate creative solutions to technical challenges. Individuals with stronger creative abilities may therefore be more capable of developing novel approaches and adapting their learning behavior when existing methods prove insufficient.

Problem solving also demonstrates a significant relationship with learning agility in established companies. Complex technological systems often generate unexpected challenges that require systematic analysis and structured solution development. Employees who possess strong problem-solving capabilities may therefore be better able to identify underlying causes of problems and develop effective strategies for addressing them. This capacity for systematic problem resolution supports the development of adaptive learning behavior.

### 5.3. Cognitive Determinants of Learning Agility in Start-Up Companies

The results for start-up companies reveal a distinctive configuration of cognitive determinants of learning agility. In this context, perception, creativity, and problem solving emerge as significant predictors, while reasoning, memory, attention, coordination, and critical thinking do not show statistically significant effects. This pattern reflects the cognitive demands of entrepreneurial environments, which are often characterized by uncertainty, rapid experimentation, and evolving organizational structures (Blank & Eckhardt, 2024).

Perceptual cognitive ability becomes particularly important in start-up environments because employees must continuously interpret ambiguous signals and identify relevant information within rapidly changing contexts. Perception allows individuals to detect patterns in environmental information and recognize cues that may indicate emerging opportunities or potential risks (Shynu et al., 2026). Employees who possess stronger perceptual abilities may therefore be more capable of responding quickly to changes in market conditions or organizational priorities. This capacity to interpret environmental signals effectively contributes to the development of learning agility in dynamic entrepreneurial settings.

Creativity again demonstrates a strong influence on learning agility in start-up companies. Entrepreneurial environments frequently encourage innovation, experimentation, and the exploration of new ideas (Andersen et al., 2022). Employees are often required to generate novel solutions and test alternative strategies in order to respond to rapidly evolving challenges. Creative cognition supports this process by enabling individuals to move beyond conventional approaches and explore new possibilities. As a result, individuals with stronger creative abilities may adapt their learning strategies more effectively when confronted with uncertainty.

Problem solving also plays a critical role in shaping learning agility within start-up companies. Many challenges encountered in entrepreneurial environments are ill-structured and lack clearly defined solutions (Amirkhizi et al., 2026). Employees must therefore rely on iterative problem-solving processes that involve testing potential solutions, evaluating outcomes, and refining strategies based on feedback. Through this process, individuals gradually develop more effective approaches to learning and adaptation. Employees who possess strong problem-solving abilities may therefore be better equipped to navigate the uncertainty inherent in start-up environments.

Interestingly, reasoning and critical thinking do not appear as significant predictors in the start-up model. This pattern may reflect the action-oriented nature of entrepreneurial organizations, where rapid experimentation and iterative learning are often prioritized over extended analytical evaluation. Instead of relying heavily on systematic analysis, employees may depend more on situational awareness, intuitive interpretation, and creative exploration when responding to emerging challenges.

The stronger influence of creativity and problem solving relative to critical thinking indicates a cognitive dynamic shaped by organizational uncertainty and structural fluidity. Entrepreneurial environments are characterized by rapid iteration cycles, evolving business models, and limited formalization, where learning frequently occurs through experimentation and direct feedback rather than through structured analytical evaluation (Blank & Eckhardt, 2024). Under such conditions, individuals must generate ideas quickly, test assumptions in real time, and implement solutions despite incomplete information. The prominence of creativity in this context reflects the necessity of divergent thinking and rapid ideation as mechanisms for navigating ambiguity.

Prior research on organizational agility suggests that organic organizational systems promote exploratory learning and adaptive experimentation, often privileging action-oriented cognition over extensive deliberation (Zhang et al., 2024). The findings of the present study empirically support this perspective by demonstrating that idea generation and solution implementation become more influential drivers of learning agility in uncertain environments. This suggests that learning agility is not a uniform capability expressed identically across contexts but rather a cognitively enacted phenomenon shaped by environmental demands. By comparing contrasting organizational structures, the study contributes to the literature by demonstrating that the cognitive determinants of agility vary systematically with structural conditions, highlighting the importance of contextualized cognition in understanding adaptive performance.

### 5.4. Contextual Differences in Learning Agility Drivers

A comparison between the two organizational contexts reveals that the cognitive foundations of learning agility are not uniform but vary according to the structural characteristics of the work environment. While creativity and problem solving consistently emerge as significant predictors across both models, other cognitive abilities demonstrate context-specific effects. In start-up companies, perception becomes an important determinant of learning agility, whereas in established companies critical thinking plays a more prominent role. These differences suggest that the cognitive processes supporting adaptive learning behavior are shaped by the nature of the tasks and decision environments encountered by employees.

Start-up environments are typically characterized by high uncertainty, evolving strategies, and limited organizational structure. Under such conditions, employees must continuously interpret ambiguous signals and respond quickly to emerging opportunities or threats. Perceptual ability therefore becomes particularly valuable because it allows individuals to recognize patterns in incomplete information and detect meaningful cues within rapidly changing environments. Employees who are capable of accurately interpreting situational signals may adapt their learning behavior more effectively because they are able to recognize when existing approaches are no longer sufficient.

In contrast, established companies often operate within structured technological systems that rely on specialized expertise, formal procedures, and data-driven decision-making. Employees working in such environments frequently encounter complex technical information that must be evaluated systematically before decisions can be made. Critical thinking therefore becomes an essential cognitive mechanism for learning agility in this context. Individuals who are capable of evaluating assumptions, assessing evidence, and interpreting technical data may develop more effective learning strategies when addressing technological challenges.

These contextual differences highlight that learning agility should not be interpreted as a single universal capability. Instead, it emerges from the interaction between individual cognitive abilities and environmental demands. Organizational environments shape the types of cognitive processes that are most useful for adapting to change. This perspective aligns with adaptive performance theory, which emphasizes that behavioral adaptability depends not only on individual capability but also on the characteristics of the environment in which individuals operate (Nandini et al., 2022).

### 5.5. Creativity as a Key Cognitive Factor Associated with Learning Agility

One of the most notable findings of this study is that creativity consistently emerges as the strongest determinant of learning agility across organizational contexts. Creativity demonstrates significant effects in the combined model as well as in both start-up and established companies’ environments, suggesting that the ability to generate novel ideas and reinterpret existing knowledge plays a central role in adaptive learning behavior.

Creativity enables individuals to approach problems from multiple perspectives and explore alternative strategies when conventional approaches prove insufficient (Nilimaa, 2023). In dynamic work environments characterized by rapid technological development and evolving organizational demands, employees frequently encounter unfamiliar situations that require flexible thinking. Individuals who possess strong creative abilities may therefore be better equipped to reinterpret past experiences, experiment with new approaches, and develop innovative solutions to emerging challenges.

From a cognitive perspective, creativity functions as a generative process that expands the range of possible responses available to an individual when confronting complex situations. This generative capability supports learning agility by encouraging experimentation, exploration, and reflective learning. Rather than relying solely on established routines, creative individuals tend to question assumptions and search for alternative interpretations of problems, which allows them to adapt their learning strategies more effectively.

The consistent importance of creativity observed in this study suggests that adaptive learning in modern workplaces may depend more strongly on generative cognitive processes than on basic information-processing efficiency. While foundational cognitive abilities such as memory and attention support task execution, creativity allows individuals to transform information into new insights and novel approaches. This transformation process appears to be a critical mechanism through which learning agility is expressed in technology-driven work environments. These findings highlight the importance of fostering creative thinking in organizational learning initiatives. Development programs that encourage idea generation, experimentation, and cross-disciplinary thinking may therefore contribute significantly to strengthening learning agility among employees.

These findings show that the relationship between cognitive abilities and learning agility is not merely a simple linear association. Instead, the relevance of specific cognitive abilities depends on the characteristics of the digital work environment. In established companies, where digital transformation is often embedded in structured systems, specialized roles, and data-based decision processes, critical thinking becomes more important because employees must evaluate technical information and make reasoned judgments. In start-up companies, where digital transformation is more closely associated with experimentation, uncertainty, and rapid iteration, perception becomes more relevant because employees must detect signals, interpret changing conditions, and respond quickly to emerging opportunities or risks. The consistent effects of creativity and problem solving across both contexts suggest that generative and solution-oriented cognition represents a core cognitive foundation of learning agility, while other cognitive abilities become more context-dependent. This pattern strengthens the originality of the study by demonstrating that learning agility is shaped by different cognitive mechanisms under different digital transformation conditions.

### 5.6. Theoretical Implications

The findings of this study contribute to the literature on learning agility by clarifying the cognitive foundations underlying adaptive learning behavior in contemporary workplaces. Previous studies have frequently conceptualized learning agility as a behavioral orientation associated with openness to experience, willingness to learn, and responsiveness to change (De Meuse, 2017; De Meuse et al., 2012). The results of the present study demonstrate that learning agility is closely associated with specific cognitive processes that enable individuals to interpret information, generate alternative perspectives, and develop effective responses to unfamiliar situations. In particular, higher-level cognitive abilities such as creativity, critical thinking, and problem solving show stronger relationships with learning agility than foundational cognitive functions such as memory, attention, or coordination.

These findings contribute to theoretical discussions on the cognitive architecture of adaptive performance. Creativity and problem solving represent generative and evaluative cognitive processes that allow individuals to transform novel situations into opportunities for learning and improvement. Creativity supports the exploration of alternative ideas and encourages experimentation, while problem solving enables individuals to structure complex situations and implement effective responses. The interaction between these processes facilitates the reinterpretation of experience and the development of adaptive learning strategies in uncertain environments.

The comparative analysis between start-up and established companies further highlights the contextual nature of learning agility. Different organizational environments activate different cognitive mechanisms that support adaptive learning behavior. Perceptual interpretation becomes more relevant in start-up environments characterized by uncertainty and rapid change, whereas analytical evaluation through critical thinking becomes more important in structured technological systems. These findings suggest that the cognitive processes underlying learning agility are shaped by the interaction between individual cognitive capabilities and environmental demands.

Taken together, the results indicate that learning agility may be understood as a cognitively grounded capability that emerges from higher-order thinking processes rather than from basic cognitive efficiency alone. This perspective extends existing research by positioning learning agility as an outcome of generative and evaluative cognitive mechanisms that enable individuals to reinterpret experience and respond adaptively to evolving work environments.

### 5.7. Managerial and Practical Implications

The results of this study provide several practical insights for organizations seeking to enhance learning agility among employees in technology-driven work environments. The findings indicate that creativity and problem-solving ability represent key cognitive capabilities associated with adaptive learning behavior. Organizations that aim to cultivate learning agility may therefore benefit from designing development programs that strengthen these higher-order cognitive skills. Training activities that encourage idea generation, collaborative problem solving, and experimentation may help employees develop more flexible approaches to learning when confronted with unfamiliar challenges.

The findings also suggest that cognitive capabilities may be considered during recruitment and talent identification processes. Assessing individuals’ capacity for creative thinking, analytical reasoning, and problem solving may help organizations identify employees who are better equipped to adapt to rapidly changing organizational conditions. Such cognitive characteristics may be particularly valuable in industries where technological change continuously reshapes work processes and skill requirements.

The observed differences between start-up and established companies further emphasize the importance of aligning human resource development strategies with the cognitive demands of the organizational environment. Start-up companies may benefit from creating work environments that encourage experimentation, rapid learning cycles, and the exploration of innovative ideas. Established companies may strengthen learning agility by fostering analytical evaluation, systematic problem solving, and evidence-based decision making. Aligning talent development initiatives with the cognitive requirements of the organizational context may therefore enhance the effectiveness of organizational learning strategies.

### 5.8. Limitations and Future Research

Several limitations should be considered when interpreting the findings of this study. The empirical data were collected from Generation Z employees working in technology-driven organizations within a single national context. Cultural factors and institutional environments may influence how cognitive abilities contribute to adaptive learning behavior. Because the study used purposive quota sampling rather than probability sampling, the sample cannot be claimed to be statistically representative of all Generation Z employees in Indonesia. The balanced sample of employees from established companies and start-up companies was appropriate for comparative analysis, but future studies using probability-based, multi-industry, or cross-national sampling would strengthen the generalizability of the findings.

Organizational context in this study was examined through comparative analysis rather than being explicitly modeled as a moderating variable in the structural framework. This methodological choice was made because the primary objective of the study was to identify and compare the cognitive determinants of learning agility within two distinct organizational settings, rather than to test a formal interaction effect between cognitive abilities and organizational context. Established companies and start-up companies were treated as separate empirical contexts because they differ in terms of structure, role formalization, task uncertainty, and learning demands. Estimating separate structural models allowed the analysis to examine which cognitive predictors emerged within each context and to compare the resulting structural patterns. Because the start-up measurement model required the removal of three learning agility indicators, the subgroup models were not fully identical at the indicator level. Therefore, testing organizational context as a formal moderating variable would require additional measurement invariance assessment before direct coefficient comparisons could be interpreted as moderation effects. Future studies may extend this work by applying multi-group analysis or moderation modeling after establishing measurement invariance across organizational groups.

The digital transformation context in this study was represented through the comparison between established companies and start-up companies, rather than through direct measurement of specific digital transformation characteristics. Therefore, the findings should be interpreted as reflecting differences between digitally intensive organizational settings, not as direct evidence of the effect of digital transformation itself. Future research may include more explicit indicators of digital transformation, such as digital maturity, technological turbulence, digital work intensity, task complexity, role fluidity, and the extent of data-driven decision making, to examine more directly how digital transformation conditions shape the relationship between cognitive abilities and learning agility.

Cognitive abilities were assessed using objective psychometric instruments, whereas learning agility was measured using self-reported behavioral indicators. Therefore, the findings should be interpreted as explaining self-reported learning agility behavior rather than objectively observed learning behavior. This approach is appropriate for capturing participants’ perceptions of their adaptive learning tendencies, but it may also be influenced by individual response bias or subjective self-assessment. Future research may incorporate supervisor ratings, peer evaluations, longitudinal learning outcomes, or behavioral performance data to provide a more comprehensive assessment of learning agility in organizational settings.

The possibility of reciprocal relationships between cognitive abilities and learning agility should also be considered. The present study conceptualized cognitive abilities as predictors of learning agility based on the theoretical assumption that cognitive resources support information processing, problem interpretation, and adaptive learning. At the same time, individuals with higher learning agility may also further develop or activate certain cognitive skills through repeated exposure to challenging experiences. Because the study used a cross-sectional design, this potential reverse relationship could not be directly tested. An instrumental variable approach was not applied because the study did not include a theoretically valid external instrument that satisfies the requirements of relevance and exogeneity. Applying an instrumental variable model without a defensible instrument could introduce additional bias rather than improve causal identification. Future research may address this issue by using longitudinal designs, cross-lagged models, experimental interventions, or instrumental variable approaches when valid instruments are available.

Further research may also explore additional psychological mechanisms that interact with cognitive abilities in shaping learning agility. Variables such as learning orientation, intrinsic motivation, resilience, and psychological safety may influence how individuals translate cognitive capability into adaptive learning behavior. Integrating cognitive, psychological, and organizational factors may therefore provide a more comprehensive explanation of learning agility in contemporary work environments.

## 6. Conclusions

This study examined the cognitive determinants of learning agility among Generation Z employees working in technology-driven organizations. By distinguishing between basic cognitive abilities and high-level cognitive abilities, the study provides a more detailed understanding of how different cognitive processes are associated with adaptive learning behavior in contemporary work environments. The findings indicate that learning agility is more strongly related to higher-order cognitive abilities than to basic cognitive efficiency. Creativity and problem solving consistently showed significant positive relationships with learning agility across the combined sample, established companies, and start-up companies, suggesting that generative thinking and solution-oriented cognition play a central role in supporting employees’ ability to learn from experience and respond to unfamiliar situations.

The results also show that some cognitive abilities are context-dependent. In established companies, critical thinking was more relevant to learning agility, reflecting the importance of analytical evaluation, evidence-based judgment, and systematic problem solving in structured technological environments. In start-up companies, perception was more strongly associated with learning agility, indicating the importance of detecting environmental cues, interpreting ambiguous information, and responding quickly to changing conditions. In contrast, memory, attention, and coordination did not show significant direct relationships with learning agility, suggesting that foundational cognitive abilities may support general task execution but are less central to adaptive learning behavior than higher-order cognitive processes.

The findings highlight learning agility as a cognitively grounded capability shaped by the interaction between individual cognitive abilities and organizational context. Rather than being driven by cognitive ability in general, learning agility appears to depend more strongly on the ability to reinterpret information, generate alternatives, and develop workable solutions under uncertainty. These findings contribute to learning agility research by clarifying the cognitive mechanisms that support adaptive learning among Generation Z employees and by showing that these mechanisms may vary across different technology-driven work environments.

## Figures and Tables

**Table 1:** Existing Studies and Research Gap.

Authors	Research Focus	Context	Method	Key Limitation/Gap
Ackerman et al. (2002)	Cognitive abilities and information processing	General workforce	Empirical study	Does not examine learning agility
F. L. Schmidt and Hunter (1998)	Cognitive ability and job performance	Employee performance	Meta-analysis	Focuses on performance outcomes rather than learning agility
Raemdonck et al. (2014)	Learning from experience and cognitive traits	Adult learners	Survey-based analysis	Limited discussion of digital workplace adaptability
Lombardo and Eichinger (2000)	Learning agility and leadership potential	Leadership development	Conceptual framework	Focused mainly on leaders and executives
De Meuse et al. (2010)	Learning agility measurement	Organizational leadership	Scale development	Treats learning agility as a general construct
Baran and Woznyj (2020)	Agility in dynamic environments	Knowledge workers	Conceptual framework	Lacks empirical testing of cognitive antecedents
Bedford (2011)	Learning agility measurement	Employees	Instrument development	Focused more on behavioral characteristics
J. Lee and Song (2022)	Integrated model and measurement of employee learning agility	General employees	Integrated literature review and scale development	Limited examination of specific cognitive determinants of learning agility

**Table 2:** Structural Model Results of Cognitive Determinants of Learning Agility Across Samples.

Variable	Combined Sample			Established Companies			Start-Up Companies		
	Coef.	*p*	f^2^	Coef.	*p*	f^2^	Coef.	*p*	f^2^
Basic Cognitive									
Reasoning	0.149	0.011 *	0.022	0.184	0.057	0.044	0.151	0.104	0.022
Memory	−0.039	0.592	0.001	−0.056	0.628	0.004	−0.093	0.390	0.006
Attention	0.093	0.138	0.009	0.167	0.086	0.027	0.074	0.364	0.006
Coordination	−0.005	0.932	0.000	0.023	0.824	0.001	−0.046	0.575	0.002
Perception	0.124	0.062	0.012	0.037	0.748	0.001	0.204	0.035 *	0.033
High-Level Cognitive									
Critical Thinking	0.142	0.016 *	0.028	0.285	0.003 **	0.132	−0.031	0.752	0.001
Creativity	0.292	0.001 ***	0.122	0.253	0.000 ***	0.127	0.371	0.001 ***	0.159
Problem Solving	0.217	0.001 ***	0.057	0.213	0.012 *	0.068	0.238	0.003 **	0.068

* *p* < 0.05, ** *p* < 0.01, *** *p* < 0.001.

**Table 3:** R^2^ and Adjusted R^2^ Results Across Samples.

	Combined Sample	Established Companies	Start-Up Companies
R^2^	0.465	0.605	0.444
Adjusted R^2^	0.448	0.580	0.409

## Data Availability

The data that support the findings of this study are available from the corresponding author upon reasonable request.

## Supplementary Materials

The following supporting information can be downloaded at: https://www.mdpi.com/article/10.3390/bs16071053/s1, Table S1. Fornell–Larcker Criterion Results for the Combined Sample; Table S2. Fornell–Larcker Results for the Fornell–Larcker Criterion Results for Established Companies; Table S3. Fornell–Larcker Results for the Fornell–Larcker Criterion Results for Start-Up Companies; Table S4. HTMT Results for the Combined Sample; Table S5. HTMT Results for the Established Companies; Table S6. HTMT Results for the Start-Up Companies.

## Appendix A

**Table A1:** Variables and Operational Definitions.

Variable Category	Variable	Conceptual Definition	Reference
Basic Cognitive Abilities	Reasoning	The logical thinking process which is inductive or deductive is used to draw conclusions from a fact.	Goel and Waechter (2017)
	Memory	The ability to store information or representations of past experiences, based on the mental processes of learning, maintenance over a period of time, and retrieval or reactivation of that memory.	Terry (2017)
	Attention	The ability to focus on relevant information while ignoring distractions	Lepsien and Nobre (2006)
	Coordination	Integration of cognitive processing with motor responses	R. C. Schmidt et al. (2011)
	Perception	The process of attaining awareness or understanding of sensory information	Qiong (2017)
High-Level Cognitive Abilities	Creativity	The mindful ability to generate a variety of novel ideas following an open-ended prompt	Olson et al. (2023)
	Critical Thinking	Ability to evaluate arguments, detect assumptions, and make evidence-based judgments	Dwyer (2017)
	Problem Solving	The ability to identify problems, search for and select various alternative solutions and determine decisions in resolving all problems faced	Bratsberg (2012)
Learning Agility	Adaptability	The ability of individuals to adapt effectively to changes in the environment and new work situations	Lombardo and Eichinger (2000)
	Applying New Learning	Ability to apply learned lessons to new situations	De Meuse et al. (2012)
	Collaborating	The ability to actively collaborate with others to create unique learning opportunities.	Hoff and Smith (2020)
	Emotional Intelligence	The ability to adapt interpersonally by demonstrating empathy, emotional control, and effective social interaction in leadership and learning contexts.	Dries and Pepermans (2012)
	Feedback Seeking	The habit of actively seeking constructive feedback on personal ideas and performance as a means of self-development.	Smith (2015)
	Mental Agility	Ability to operate effectively in complex situations, to view problems from different perspectives, and to respond appropriately in novel and uncertain environments.	Lombardo and Eichinger (2000)
	Information Seeking	The habit of continuously seeking and updating information and knowledge to remain relevant and competent.	Lombardo and Eichinger (2000)
	People Agility	Ability to work effectively with diverse individuals, supported by strong self-awareness and the capacity to interact with others in a constructive manner.	Lombardo and Eichinger (2000)
	Performance Agility	Ability to achieve expected outcomes in uncertain or first-time situations, even when confronted with new and challenging conditions.	Lombardo and Eichinger (2000)
	Critical Reflection	The habit of actively evaluating one’s own behavior and performance critically to encourage continuous improvement and learning.	Mitchinson et al. (2012)
	Self-Awareness	The ability to recognize personal strengths and weaknesses as a basis for self-growth and development.	De Meuse et al. (2012)
	Self-Directed Learning	Proactively seeking out learning experiences	J. Lee and Song (2022)
	Willingness to Learn	Motivation and openness to learn new skills	Im et al. (2017)

**Table A2:** Learning Agility Measurement Items.

Dimension	Code	Item	Reference
Adaptability	LA ADP1	I can quickly adapt when there are changes in the workplace.	Pulakos et al. (2000)
	LA ADP2	I feel comfortable working in situations I have never experienced before.	LePine et al. (2000)
	LA ADP3	I can quickly adjust my work priorities when sudden changes occur.	Griffin and Hesketh (2003)
Applying New Learning	LA ANL1	I often apply previous experiences to complete new tasks.	DeRue et al. (2012)
	LA ANL2	I set my learning goals independently.	García et al. (2024)
	LA ANL3	I am pleased to practice the knowledge.	S. Wang et al. (2021)
Collaborating	LA CLB1	I enjoy collaborating in a team to complete tasks.	Pulakos et al. (2002)
	LA CLB2	I am open to ideas from colleagues to achieve the best results.	Bouland-van Dam et al. (2022)
	LA CLB3	I can resolve differences of opinion within a team constructively to achieve shared goals.	De Dreu and Weingart (2003)
Emotional Intelligence	LA EMI1	I am able to control my emotions in stressful work situations.	Ployhart and Bliese (2006)
	LA EMI2	I can understand other people’s feelings while working in a team.	Salovey et al. (2003)
	LA EMI3	I can use positive emotions to enhance my motivation and job performance	Mayer et al. (2008)
Feedback Seeking	LA FS1	I actively seek feedback from my supervisor regarding my performance.	Nae et al. (2015)
	LA FS2	I ask for feedback from colleagues to improve work results.	London (2014)
	LA FS3	I independently monitor my work performance to evaluate whether it meets expected standards	Anseel et al. (2015)
Mental Agility	LA MA1	I can think flexibly when facing sudden changes.	Karman (2020)
	LA MA2	I can solve problems from different perspectives.	Van Merrienboer (2013)
	LA MA3	I can quickly change my approach when the initial solution does not work.	Pulakos et al. (2002)
Information Seeking	LA IFS1	I actively seek new information to support my work.	Shah (2014)
	LA IFS2	I use various sources to deepen my understanding of tasks.	Cerasoli et al. (2014)
	LA IFS3	I am confident of getting useful information even if there is no one around to show me how to do it.	F. L. Cook et al. (2010)
People Agility	LA PA1	I can adjust my communication style with different types of colleagues.	Luo et al. (2016)
	LA PA2	I am able to build positive work relationships with different types of people.	Kahn (2017)
	LA PA3	I easily adapt when working with people from diverse backgrounds.	Sam and Berry (2010)
Performance Agility	LA PFA1	I remain productive even when working under time pressure.	Gusy et al. (2021)
	LA PFA2	I am task-oriented, completing assignments on time and efficiently.	Kao (2017)
	LA PFA3	I stay focused on achieving results even when facing uncertainty.	Tseng and Kang (2008)
Critical Reflection	LA CTR1	I evaluate my work methods after completing tasks.	Claessens et al. (2010)
	LA CTR2	I learn from the mistakes I have made.	Kwok and Kwong (2023)
	LA CTR3	I identify aspects of my work process that most hinder my performance.	Salanova et al. (2010)
Self-awareness	LA SAW1	I am aware of my strengths and weaknesses at work.	Sutton et al. (2015)
	LA SAW2	I reflect on how my behavior affects others in the workplace.	Eurich (2018)
	LA SAW3	I recognize my emotions and how they influence my work performance.	Moon and Hur (2011)
Self-directed Learning	LA SDL1	I take the initiative to identify my learning needs.	Hetzner et al. (2012)
	LA SDL2	I set personal goals for my learning and development.	Travers et al. (2015)
	LA SDL3	I actively search for resources to improve my skills.	Van den Heuvel et al. (2015)
Willingness to Learn	LA WTL1	I view challenges as opportunities to grow and learn.	Blackwell et al. (2007)
	LA WTL2	I am motivated to continuously improve my knowledge and abilities.	D. A. Cook and Artino (2016)
	LA WTL3	I persist in learning new things even when the process is challenging and uncertain.	Overoye and Storm (2015)
