# Supplementary material for: The Influence of Different Cognitive Skills on Learning Agility Among Gen Z in Established and Start-Up Companies
Source: Behav Sci (Basel). 2026 Jun 24;16(7):1053. doi: 10.3390/bs16071053 (PMC13404940; doi:10.3390/bs16071053)
Supplement: Supplementary file 1 [file behavsci-16-01053-s001.zip › behavsci-4341933-supplementary.pdf]

Supplementary Materials

Table S1. Fornell–Larcker Criterion Results for the Combined Sample

	BC_AT	BC_CR	BC_MM	BC_PC	BC_RS	HC_CR	HC_CT	HC_PS	LA_ADP	LA_ANL	LA_CLB	LA_CTR	LA_EMI	LA_FS	LA_IFS	LA_MA	LA_PA	LA_PFA	LA_SAW	LA_SDL	LA_WTL
BC_AT	**1,000**																				
BC_CR	0,511	**1,000**																			
BC_MM	0,520	0,500	**1,000**																		
BC_PC	0,548	0,547	0,640	**1,000**																	
BC_RS	0,494	0,414	0,600	0,582	**1,000**																
HC_CR	0,358	0,366	0,327	0,331	0,328	**1,000**															
HC_CT	0,336	0,290	0,286	0,331	0,263	0,333	**1,000**														
HC_PS	0,370	0,445	0,344	0,491	0,263	0,308	0,414	**1,000**													
LA_ADP	0,463	0,375	0,342	0,427	0,358	0,443	0,386	0,437	**0,855**												
LA_ANL	0,325	0,269	0,245	0,300	0,324	0,414	0,203	0,273	0,540	**0,839**											
LA_CLB	0,279	0,316	0,282	0,403	0,325	0,374	0,396	0,321	0,580	0,512	**0,881**										
LA_CTR	0,377	0,384	0,336	0,435	0,353	0,418	0,286	0,389	0,537	0,554	0,526	**0,829**									
LA_EMI	0,328	0,314	0,305	0,378	0,310	0,445	0,411	0,450	0,583	0,532	0,646	0,571	**0,843**								
LA_FS	0,382	0,319	0,310	0,410	0,348	0,349	0,349	0,426	0,605	0,546	0,590	0,614	0,569	**0,865**							
LA_IFS	0,372	0,356	0,290	0,346	0,381	0,381	0,341	0,324	0,549	0,559	0,509	0,552	0,600	0,531	**0,833**						
LA_MA	0,306	0,220	0,269	0,339	0,261	0,325	0,263	0,415	0,589	0,551	0,528	0,507	0,544	0,617	0,524	**0,846**					
LA_PA	0,394	0,383	0,314	0,392	0,321	0,495	0,282	0,414	0,540	0,594	0,481	0,574	0,552	0,605	0,613	0,589	**0,840**				
LA_PFA	0,280	0,195	0,302	0,380	0,323	0,339	0,341	0,356	0,540	0,499	0,640	0,590	0,626	0,678	0,526	0,599	0,547	**0,846**			
LA_SAW	0,281	0,258	0,286	0,318	0,313	0,415	0,381	0,336	0,509	0,527	0,550	0,497	0,592	0,620	0,524	0,540	0,512	0,600	**0,859**		
LA_SDL	0,331	0,314	0,278	0,379	0,363	0,344	0,288	0,335	0,516	0,516	0,515	0,483	0,497	0,558	0,628	0,559	0,627	0,586	0,537	**0,845**	
LA_WTL	0,306	0,254	0,287	0,299	0,325	0,392	0,310	0,334	0,503	0,474	0,449	0,497	0,534	0,549	0,521	0,541	0,420	0,531	0,484	0,517	**0,816**

Table S2. Fornell–Larcker Results for the Fornell–Larcker Criterion Results for Established Companies

	BC_AT	BC_CR	BC_MM	BC_PC	BC_RS	HC_CR	HC_CT	HC_PS	LA_ADP	LA_ANL	LA_CLB	LA_CTR	LA_EMI	LA_FS	LA_IFS	LA_MA	LA_PA	LA_PFA	LA_SAW	LA_SDL	LA_WTL
BC_AT	**1,000**																				
BC_CR	0,698	**1,000**																			
BC_MM	0,613	0,578	**1,000**																		
BC_PC	0,687	0,602	0,621	**1,000**																	
BC_RS	0,558	0,525	0,547	0,633	**1,000**																
HC_CR	0,253	0,293	0,184	0,247	0,312	**1,000**															
HC_CT	0,328	0,260	0,364	0,389	0,283	0,375	**1,000**														
HC_PS	0,389	0,462	0,370	0,496	0,290	0,286	0,495	**1,000**													
LA_ADP	0,486	0,438	0,359	0,410	0,368	0,388	0,467	0,425	**0,842**												
LA_ANL	0,367	0,355	0,168	0,275	0,312	0,484	0,336	0,334	0,551	**0,839**											
LA_CLB	0,362	0,370	0,336	0,425	0,432	0,408	0,557	0,368	0,626	0,539	**0,865**										
LA_CTR	0,475	0,498	0,393	0,473	0,433	0,395	0,349	0,380	0,496	0,624	0,511	**0,840**									
LA_EMI	0,394	0,362	0,400	0,361	0,402	0,421	0,557	0,500	0,591	0,607	0,702	0,522	**0,839**								
LA_FS	0,445	0,328	0,282	0,456	0,374	0,375	0,459	0,541	0,641	0,546	0,530	0,602	0,564	**0,850**							
LA_IFS	0,467	0,458	0,405	0,459	0,553	0,431	0,466	0,326	0,533	0,545	0,557	0,617	0,559	0,498	**0,809**						
LA_MA	0,353	0,280	0,306	0,386	0,284	0,313	0,470	0,545	0,545	0,458	0,512	0,488	0,561	0,641	0,442	**0,847**					

LA_PA	0,453	0,458	0,287	0,394	0,317	0,564	0,346	0,411	0,456	0,631	0,442	0,609	0,524	0,570	0,581	0,569	**0,843**				
LA_PFA	0,337	0,284	0,342	0,451	0,385	0,333	0,484	0,435	0,523	0,452	0,719	0,583	0,652	0,676	0,438	0,573	0,502	**0,849**			
LA_SAW	0,324	0,189	0,235	0,321	0,348	0,374	0,478	0,399	0,496	0,558	0,648	0,460	0,659	0,671	0,559	0,578	0,561	0,659	**0,849**		
LA_SDL	0,386	0,378	0,296	0,476	0,451	0,371	0,499	0,384	0,525	0,452	0,576	0,500	0,503	0,514	0,572	0,528	0,571	0,601	0,594	**0,833**	
LA_WTL	0,298	0,333	0,296	0,348	0,364	0,396	0,395	0,440	0,555	0,451	0,466	0,545	0,515	0,585	0,472	0,574	0,409	0,495	0,471	0,518	**0,793**

Table S3. Fornell–Larcker Results for the Fornell–Larcker Criterion Results for Start-Up Companies

	BC_AT	BC_CR	BC_MM	BC_PC	BC_RS	HC_CR	HC_CT	HC_PS	LA_ADP	LA_ANL	LA_CLB	LA_EMI	LA_FS	LA_IFS	LA_MA	LA_PA	LA_PFA	LA_SDL
BC_AT	**1,000**																	
BC_CR	0,325	**1,000**																
BC_MM	0,426	0,422	**1,000**															
BC_PC	0,410	0,492	0,660	**1,000**														
BC_RS	0,429	0,303	0,654	0,532	**1,000**													
HC_CR	0,462	0,438	0,470	0,415	0,345	**1,000**												
HC_CT	0,344	0,320	0,208	0,274	0,244	0,291	**1,000**											
HC_PS	0,351	0,428	0,318	0,486	0,236	0,330	0,332	**1,000**										
LA_ADP	0,449	0,312	0,333	0,458	0,357	0,518	0,305	0,463	**0,867**									
LA_ANL	0,292	0,175	0,355	0,350	0,358	0,353	0,047	0,209	0,497	**0,832**								
LA_CLB	0,196	0,269	0,230	0,396	0,220	0,359	0,235	0,287	0,498	0,439	**0,896**							
LA_EMI	0,279	0,286	0,219	0,425	0,229	0,505	0,272	0,425	0,545	0,369	0,540	**0,837**						
LA_FS	0,409	0,405	0,465	0,474	0,423	0,435	0,288	0,379	0,562	0,485	0,680	0,498	**0,832**					
LA_IFS	0,293	0,266	0,175	0,242	0,204	0,362	0,221	0,359	0,539	0,527	0,382	0,594	0,430	**0,849**				
LA_MA	0,268	0,159	0,242	0,304	0,251	0,372	0,000	0,278	0,635	0,663	0,512	0,456	0,486	0,584	**0,828**			
LA_PA	0,365	0,323	0,383	0,430	0,355	0,452	0,232	0,455	0,633	0,504	0,483	0,520	0,567	0,588	0,556	**0,815**		
LA_PFA	0,256	0,094	0,309	0,355	0,298	0,433	0,186	0,309	0,560	0,531	0,466	0,516	0,514	0,573	0,572	0,532	**0,794**	
LA_SDL	0,300	0,262	0,296	0,287	0,285	0,355	0,015	0,314	0,480	0,583	0,365	0,397	0,509	0,644	0,549	0,651	0,427	**0,840**

Table S4. HTMT Results for the Combined Sample

	BC_AT	BC_CR	BC_MM	BC_PC	BC_RS	HC_CR	HC_CT	HC_PS	LA_ADP	LA_ANL	LA_CLB	LA_CTR	LA_EMI	LA_FS	LA_IFS	LA_MA	LA_PA	LA_PFA	LA_SAW	LA_SDL	LA_WTL
BC_AT																					
BC_CR	0,511																				
BC_MM	0,520	0,500																			
BC_PC	0,548	0,547	0,640																		
BC_RS	0,494	0,414	0,600	0,582																	
HC_CR	0,358	0,366	0,327	0,331	0,328																
HC_CT	0,336	0,290	0,286	0,331	0,263	0,333															
HC_PS	0,370	0,445	0,344	0,491	0,263	0,308	0,414														
LA_ADP	0,513	0,415	0,378	0,473	0,396	0,490	0,427	0,484													
LA_ANL	0,366	0,302	0,276	0,338	0,366	0,467	0,229	0,307	0,672												
LA_CLB	0,301	0,342	0,304	0,436	0,349	0,404	0,428	0,348	0,694	0,622											
LA_CTR	0,428	0,436	0,381	0,493	0,402	0,476	0,326	0,442	0,675	0,707	0,642										
LA_EMI	0,369	0,354	0,343	0,424	0,348	0,499	0,461	0,506	0,723	0,667	0,783	0,725									
LA_FS	0,419	0,350	0,340	0,451	0,382	0,381	0,381	0,467	0,732	0,672	0,696	0,764	0,696								
LA_IFS	0,421	0,403	0,329	0,393	0,431	0,430	0,385	0,365	0,687	0,711	0,620	0,710	0,760	0,657							

LA_MA	0,341	0,245	0,301	0,377	0,291	0,360	0,292	0,463	0,725	0,692	0,633	0,640	0,675	0,754	0,659						
LA_PA	0,443	0,430	0,353	0,441	0,361	0,557	0,317	0,465	0,672	0,752	0,582	0,734	0,692	0,746	0,780	0,739					
LA_PFA	0,311	0,218	0,336	0,425	0,361	0,378	0,381	0,399	0,666	0,625	0,770	0,749	0,782	0,830	0,664	0,746	0,688				
LA_SAW	0,309	0,286	0,316	0,350	0,345	0,458	0,420	0,370	0,620	0,653	0,652	0,622	0,728	0,749	0,651	0,662	0,633	0,737			
LA_SDL	0,371	0,351	0,311	0,427	0,408	0,383	0,320	0,373	0,639	0,649	0,614	0,614	0,619	0,681	0,798	0,695	0,789	0,727	0,659		
LA_WTL	0,352	0,290	0,332	0,344	0,374	0,449	0,359	0,387	0,640	0,614	0,554	0,652	0,690	0,695	0,675	0,692	0,545	0,685	0,618	0,662	

Table S5. HTMT Results for the Established Companies

	BC_AT	BC_CR	BC_MM	BC_PC	BC_RS	HC_CR	HC_CT	HC_PS	LA_ADP	LA_ANL	LA_CLB	LA_CTR	LA_EMI	LA_FS	LA_IFS	LA_MA	LA_PA	LA_PFA	LA_SAW	LA_SDL	LA_WTL
BC_AT																					
BC_CR	0,698																				
BC_MM	0,613	0,578																			
BC_PC	0,687	0,602	0,621																		
BC_RS	0,558	0,525	0,547	0,633																	
HC_CR	0,253	0,293	0,184	0,247	0,312																
HC_CT	0,328	0,260	0,364	0,389	0,283	0,375															
HC_PS	0,389	0,462	0,370	0,496	0,290	0,286	0,495														
LA_ADP	0,545	0,492	0,402	0,460	0,412	0,432	0,524	0,476													
LA_ANL	0,413	0,399	0,188	0,310	0,352	0,545	0,378	0,376	0,694												
LA_CLB	0,400	0,407	0,368	0,466	0,469	0,442	0,607	0,401	0,768	0,658											
LA_CTR	0,533	0,559	0,439	0,530	0,487	0,444	0,391	0,428	0,623	0,788	0,620										
LA_EMI	0,445	0,411	0,454	0,406	0,453	0,473	0,629	0,564	0,742	0,765	0,864	0,658									
LA_FS	0,494	0,365	0,313	0,505	0,416	0,417	0,509	0,601	0,799	0,683	0,641	0,751	0,705								
LA_IFS	0,544	0,534	0,474	0,537	0,645	0,501	0,540	0,378	0,694	0,712	0,702	0,808	0,732	0,644							
LA_MA	0,396	0,313	0,342	0,430	0,318	0,346	0,524	0,603	0,678	0,578	0,617	0,608	0,696	0,793	0,570						
LA_PA	0,506	0,512	0,321	0,442	0,358	0,634	0,385	0,461	0,571	0,792	0,534	0,765	0,654	0,712	0,757	0,713					
LA_PFA	0,375	0,317	0,380	0,502	0,429	0,371	0,538	0,486	0,649	0,566	0,876	0,731	0,816	0,839	0,567	0,712	0,627				
LA_SAW	0,360	0,210	0,261	0,357	0,388	0,416	0,532	0,442	0,617	0,700	0,783	0,576	0,823	0,831	0,725	0,717	0,700	0,818			
LA_SDL	0,439	0,429	0,333	0,547	0,514	0,418	0,562	0,428	0,659	0,571	0,693	0,636	0,636	0,637	0,760	0,653	0,725	0,745	0,744		
LA_WTL	0,354	0,395	0,352	0,414	0,434	0,471	0,471	0,525	0,738	0,604	0,600	0,729	0,691	0,774	0,655	0,758	0,545	0,658	0,625	0,691	

Table S6. HTMT Results for the Start-Up Companies

	BC_AT	BC_CR	BC_MM	BC_PC	BC_RS	HC_CR	HC_CT	HC_PS	LA_ADP	LA_ANL	LA_CLB	LA_EMI	LA_FS	LA_IFS	LA_MA	LA_PA	LA_PFA	LA_SDL
BC_AT																		
BC_CR	0,325																	
BC_MM	0,426	0,422																
BC_PC	0,410	0,492	0,660															
BC_RS	0,429	0,303	0,654	0,532														
HC_CR	0,462	0,438	0,470	0,415	0,345													
HC_CT	0,344	0,320	0,208	0,274	0,244	0,291												
HC_PS	0,351	0,428	0,318	0,486	0,236	0,330	0,332											
LA_ADP	0,492	0,342	0,364	0,501	0,390	0,567	0,335	0,507										
LA_ANL	0,331	0,198	0,405	0,395	0,408	0,400	0,056	0,236	0,614									
LA_CLB	0,205	0,286	0,246	0,423	0,231	0,381	0,247	0,304	0,578	0,528								
LA_EMI	0,316	0,323	0,247	0,480	0,259	0,570	0,306	0,481	0,673	0,470	0,647							

LA_FS	0,465	0,463	0,523	0,540	0,480	0,482	0,326	0,434	0,692	0,623	0,820	0,632					
LA_IFS	0,326	0,297	0,195	0,270	0,227	0,403	0,245	0,399	0,658	0,664	0,454	0,746	0,538				
LA_MA	0,304	0,180	0,276	0,345	0,286	0,422	0,067	0,321	0,793	0,849	0,614	0,583	0,622	0,741			
LA_PA	0,419	0,374	0,442	0,497	0,409	0,524	0,266	0,524	0,802	0,655	0,594	0,679	0,737	0,754	0,731		
LA_PFA	0,300	0,112	0,366	0,423	0,355	0,514	0,222	0,370	0,732	0,708	0,594	0,697	0,690	0,759	0,769	0,727	
LA_SDL	0,337	0,296	0,334	0,324	0,322	0,400	0,029	0,355	0,592	0,742	0,437	0,505	0,638	0,808	0,707	0,844	0,564
